# Establishment of an Efficient Immortalization Strategy Using HMEJ-Based b*TERT* Insertion for Bovine Cells

**DOI:** 10.3390/ijms222212540

**Published:** 2021-11-21

**Authors:** Zihan Zhang, Zhuo Han, Ying Guo, Xin Liu, Yuanpeng Gao, Yong Zhang

**Affiliations:** 1 College of Veterinary Medicine, Northwest A&F University, Xianyang 712100, China; zzh1993@nwsuaf.edu.cn (Z.Z.); hz1991@nwsuaf.edu.cn (Z.H.); guoying@nwafu.edu.cn (Y.G.); Liu_Xin@nwafu.edu.cn (X.L.); 2 Key Laboratory of Animal Biotechnology, Ministry of Agriculture and Rural Affairs, Northwest A&F University, Xianyang 712100, China

**Keywords:** immortalization, bovine, telomerase reverse transcriptase (*TERT*), homology-mediated end joining (HMEJ), CRISPR/Cas9, macrophage

## Abstract

Immortalized cell lines have been used in a wide range of applications in research on immune disorders and cellular metabolic regulation due to the stability and uniformity of their cellular characteristics. At present, the investigation into molecular functions and signaling pathways within bovine cells remains largely limited by the lack of immortalized model cells. Current methods for immortalizing bovine cells are mainly restricted to the ectopic expression of human telomerase reverse transcriptase (hTERT) through transient transfection or virus-mediated delivery, which have defects in efficiency and reliability. In this study, we identified bovine TERT (bTERT) as a novel potent biofactor for immortalizing bovine cells with great advantages over hTERT, and established an efficient and easily manipulated strategy for the immortalization of bovine primary cells. Through the homology-mediated end-joining-based insertion of bTERT at the ROSA26 locus, we successfully generated immortalized bovine fetal fibroblast cell lines with stable characteristics. The observed limitation of this strategy in immortalizing bovine bone marrow-derived macrophages was attributed to the post-translational modification of bTERT, causing inhibited nuclear localization and depressed activity of bTERT in this terminally differentiated cell. In summary, we constructed an innovative method to achieve the high-quality immortalization of bovine primary cells, thereby expanding the prospects for the future application of immortalized bovine model cell lines.

## 1. Introduction

Immortalized cell lines play an important role in the functional gene research and cell signaling pathway of cattle [1,2,3,4]. Given the lack of bovine immortalized cell lines, most related studies on cattle only use human or mouse cell lines as model cells, which may hinder subsequent research and application [5,6]. The traditional immortalization strategies for bovine cells generally introduce human telomerase reverse transcriptase (h*TERT*) genes, oncogenes, or viral genomes into primary cells via transient transfection or a virus-mediated delivery system and construct long-term cultured cell lines in vitro [7]. However, there are some limitations and risks in the subsequent use of cell lines generated via these methods, such as the inefficiency of the immortalized cell lines caused by the short expression time of the immortalization factor via transient transfection, as well as the instability of the cell genome because of the integration of random exogenous immortalization factors through virus-mediated delivery [8,9,10,11,12,13]. Additionally, there are unknown obstacles for the immortalization of bovine cells. Thus, an efficient and easily manipulated immortalized strategy exclusive for bovine cells is necessary.

The CRISPR/Cas9 system, a genome precise editing technology, can achieve precise knockout and insertion, which has been widely used in livestock breeding and selection [14,15,16]. Recently, CRISPR/Cas9 also has applications in immortalizing human and mouse cells [17,18]. TERT, the catalytic subunit of the telomerase complex, is a positive regulator of telomerase. At present, research on the function of human TERT (hTERT) is profound, and the application of hTERT in immortalization is pervasive at the same time. Nevertheless, porcine TERT can be used to immortalize porcine macrophages, and chicken TERT can immortalize chicken preadipocytes [19,20]. Even so, the application of bovine TERT (bTERT) in the establishment of immortalized cell lines has not been reported.

The highlight of this study is that it is the first to identify bTERT as an efficient factor for immortalization with obvious advantages over hTERT in improving telomerase activity and maintaining telomere length in bovine cells. Therefore, we performed a homology-mediated end joining (HMEJ)-based CRISPR/Cas9-mediated site-specific insertion of bTERT at the bovine ROS26 (bROSA26) locus and realized the immortalization of bovine fetal fibroblasts (BFFs) with high efficiency and stable characteristics. Furthermore, we revealed that the endogenous hindrance of this strategy in promoting the immortalization of bovine bone marrow-derived macrophages was mainly reflected in the blockage of nuclear translocation and inhibition of enzymatic activity of bTERT regulated by post-translational modification. Focusing on bovine primary cells, we established a practical immortalization approach with future application potential by eliminating the inhibitory regulation of bTERT in terminally differentiated cells.

## 2. Results

### 2.1. bTERT Is a Potent Factor for Immortalization

At present, h*TERT* is a typical factor for the immortalization of bovine cells [4,5,6,7,8,9,10,11,12,13,14,15,16,17,18,19,20,21]. However, the ectopic expression of hTERT has unknown effects due to the introduction of genes from heterologous animals. Therefore, we expected to use b*TERT* to achieve the immortalization of bovine cells. First, we found that the protein conservation between bTERT and hTERT was higher than bTERT and goat or mouse TERT (Figure S1), implying that bTERT and hTERT may be similar in function. Second, we explored the distribution characteristics of bTERT expression in the main tissues of the organism and compared them with hTERT. Quantitative real-time PCR (qPCR) and Western blot analysis showed that bTERT was mainly expressed in the ovary and testis (Figure S2), which resembled the expression pattern of hTERT [22,23]. RNA binding protein immunoprecipitation assay and semi-quantitative PCR experiment revealed that bTERT interacted with TERCs in mouse embryonic fibroblasts (MEFs), goat fetal fibroblasts (GFFs), and HEK-293T cells. Both hTERT and mTERT only interacted with hTERC and mTERC in HEK-293T and MEFs [24], respectively (Figure S3), indicating that bTERT could adapt and constitute with TERCs of multiple species.

**Figure 1:**
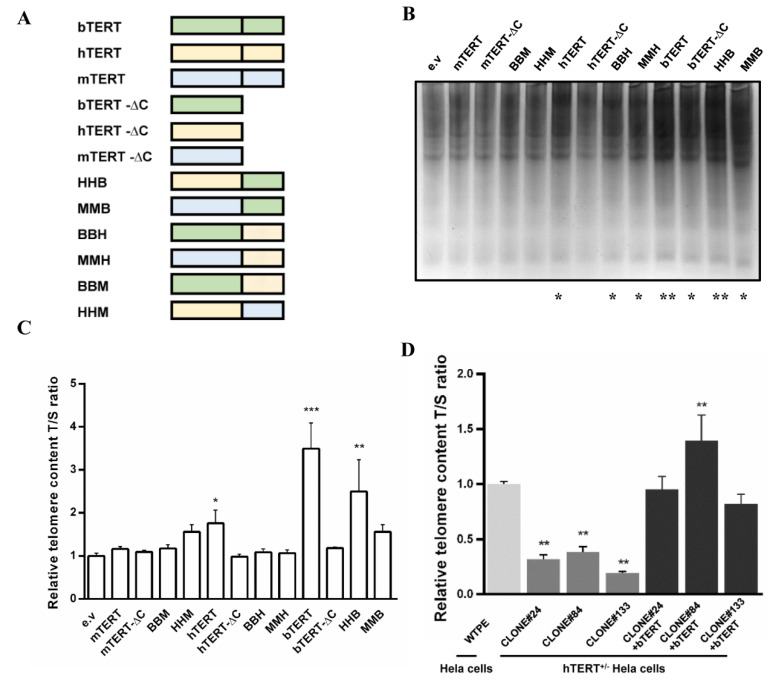
b*TERT* is a potent factor for immortalization. (**A**) Diagram of expression vectors of *TERT*s. TERTs₋−∆C represent truncated expression vectors without CTD domain. HHB, MMB, BBH, MMH, BBM, and HHM represent the substitute recombinant expression vectors with CTD replacement of TERTs of different species. Green is for bovine TERT, yellow is for human TERT, and blue is for mouse TERT. (**B**) TRAP analysis of BFFs transfected with *TERT*s, TERTs₋−∆C, substitute recombinant *TERT*s or empty vector. e.v, empty vector. (**C**) Relative telomere length of BFFs transfected with *TERT*s. TERTs₋−∆C, substitute recombinant *TERT*s or empty vector, respectively. Bovine β₋globin served as internal reference. e.v., empty vector. (**D**) Relative telomere length of WTPE, three h*TERT* ^+/−^ colonies of Hela cells (CLONE#24, CLONE#84, and CLONE#133), and three h*TERT* ^+/−^ colonies resupplied with bTERT. “WTPE” represents wild₋type Hela cells. Human β₋globin served as internal reference. Data in B₋D were mean ± s.d., *n* = 3 independent experiments, one₋tailed Student’s *t*₋test. * *p* < 0.05, ** *p* < 0.01, *** *p* < 0.001 vs. e.v or WTPE.

Previous studies found that the C terminal extension analogous to a polymerase thumb domain (CTD) of h*TERT* is essential for cell immortalization [24,25]. In view of this, we focused on the effect of the CTD of b*TERT.* According to Huard et al. [25], we used h*TERT* as the reference and defined the amino acid residue 936 of h*TERT* as the starting position of the CTD (Figure S1), while mTert was used as the species control. As shown in Figure 1A, truncated expression vectors without CTD (bTERT-∆C, hTERT-∆C, and mTERT-∆C) and substitute recombinant expression vectors (BBH, BBM, HHB, HHM, MMH, and MMB) with CTD replacement of TERT of different species were constructed, while three different species of primary cells (BFFs, GFFs, and MEFs) were used as the study subjects. We found that the relative telomere length of BFFs and GFFs was significantly shorter than that of MEFs (Figure S4), and the function of bTERT was stronger than that of hTERT and mTERT in increasing telomerase activity. Only bTERT significantly prolonged the telomere length in these three cell types (Figure 1B,C, Figures S5 and S6), which suggested that bTERT performed more effectively than hTERT in primary cells, especially in bovine and goat primary cells. Moreover, the promotion effect of bTERT-∆C on telomerase activity was weaker than that of wild-type bTERT, and bTERT-∆C almost lost the ability to lengthen telomeres (Figure 1B,C, Figures S5 and S6), indicating that the CTD is essential for TERT to function normally. The promotion of telomerase activity by BBH and BBM significantly reduced compared with that by wild-type bTERT in BFFs, and neither BBH nor BBM could prolong telomere in the primary cells of all three species (Figure 1B,C, Figures S5 and S6). Interestingly, we found that HHB and MMB had better effects on telomerase activity than wild-type hTERT or mTERT in BFFs and MEFs (Figure 1B and Figure S6A). Compared with hTERT-∆C and mTERT-∆C, HHB and MMB significantly extended the telomere length (Figure 1C, Figures S5B and S6B), implying that the CTD of bTERT has an advantage in promoting telomere elongation. In the primary cells of domestic animals, bTERT has a stronger ability to promote telomerase activity and extend telomere length than hTERT (Table 1).

Subsequently, we explored whether bTERT can replace endogenous hTERT to maintain cell proliferation. To this end, we constructed *TERT*^+/−^ (referred to as “hTERT haploinsufficiency” interchangeably hereafter) knockout colonies of Hela cells using the CRISPR/Cas9 system (Figure S7). However, we did not obtain *TERT* homozygous knockout colonies, consistent with the research of Wen et al. [26]. Subsequently, we resupplied b*TERT* into *TERT*^+/−^ Hela cells and then detected the proliferation activity, telomerase activity assay, and relative telomere length. After resupplying bTERT, the above three aspects improved to normal levels of wild-type Hela cells compared with *TERT*^+/−^ Hela cells (Figure 1D and Figure S8). The results indicated that bTERT could compensate for the function of endogenous hTERT in Hela cells.

These results demonstrated that bTERT could replace the role of hTERT in promoting cell survival by maintaining the telomerase activity and relative telomere length of cells due to the high conservation with hTERT in protein sequence, structure, and function. bTERT is more suitable for the immortalization of bovine cells than hTERT. Therefore, we chose b*TERT* as a novel immortalization factor for establishing immortalized bovine cell lines.

### 2.2. HMEJ-Based Site-Specific bTERT Insertion Enables Efficient Immortalization of Bovine Cells

**Figure 2:**
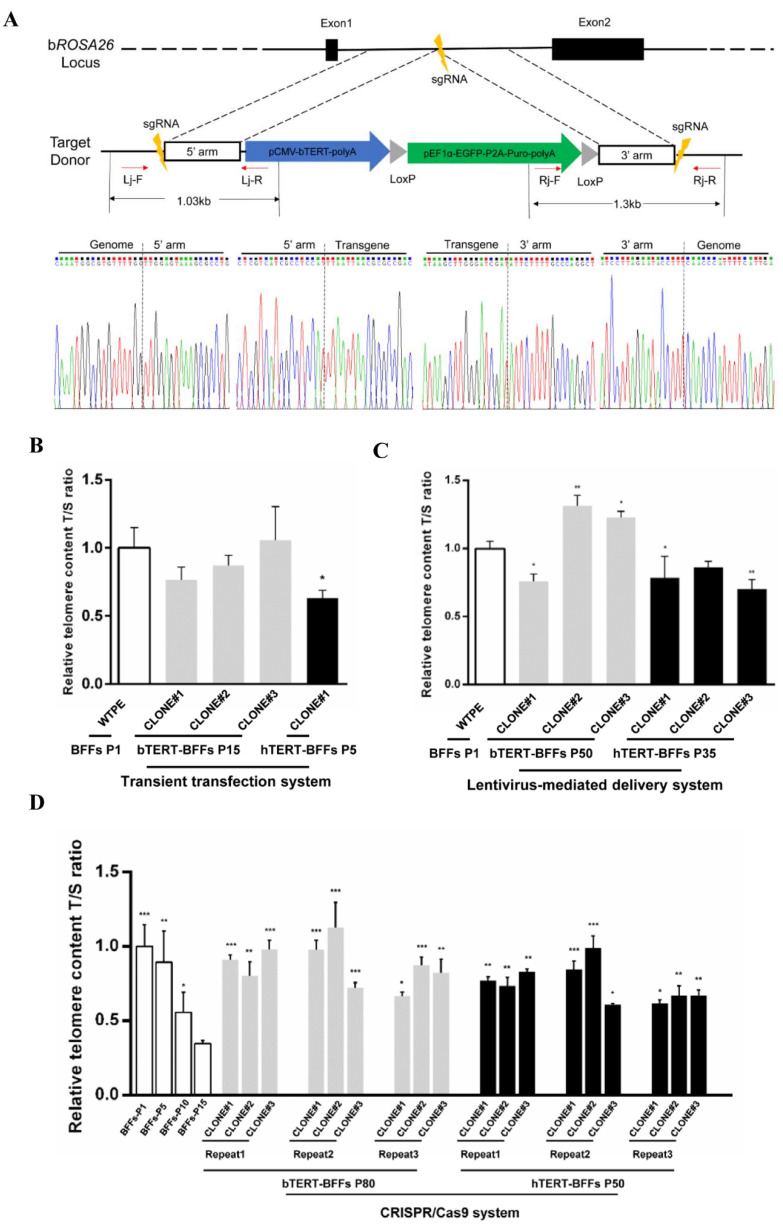
Immortalization strategy of HMEJ-based CRISPR/Cas9-mediated b*TERT* insertion at the b*ROSA26* locus has great advantages in efficiency and cell viability. (**A**) Schematic overview of the screening of the individual colonies via HMEJ-based b*TERT* insertion at the b*ROSA26* locus. Lj-F/Lj-R, 5′ junction PCR primer; Rj-F/Rj-R, 3′ junction PCR primer. Sanger sequencing confirmed the precise insertion of exogenous DNA. (**B**) Relative telomere length of wild-type 1st passage of BFFs, 15th passage of BFFs via transient transfection with b*TERT*, and 5th passage of BFFs via transient transfection with h*TERT*. “WTPE” represents wild-type BFFs. Bovine β-globin served as internal reference. (**C**) Relative telomere length of immortalized BFFs via lentivirus-mediated delivery with b*TERT* or h*TERT*. Bovine β-globin served as internal reference. (**D**) Relative telomere length of immortalized BFFs colonies integrated with b*TERT* or h*TERT* by the CRISPR/Cas9 system. Bovine β-globin served as internal reference. Data in (**B**–**D**) were mean ± s.d., *n* = 3 independent experiments, one-tailed Student’s *t*-test, * *p* < 0.05, ** *p* < 0.01, *** *p* < 0.001 vs. BFFs P1 (**B**,**C**) or BFFs P15 (**D**).

Here, we expressed bTERT stably at the b*ROSA26* locus by the HMEJ-based CRISPR/Cas9 system in BFFs to explore the ability to establish immortalized BFFs and create a novel and efficient strategy to realize the immortalization of bovine primary cells. First, by analyzing the sequence characteristics of the b*ROSA26* locus, we designed three sgRNAs specific to the b*ROSA26* locus intron 1 region. After a comprehensive evaluation of the activities and potential off-target sites of the three sgRNAs, we selected target site 3 for the insertion of the exogenous gene (Figure 2A). Subsequently, plasmid encoding Cas9 protein, Cas9/sgRNA3, was cotransfected with the donor vector pCMV-b*TERT*-pEF1α-EGFP-PURO-HMEJ into BFFs and achieved the insertion of the b*TERT* gene. After screening with puromycin, drug-resistant colonies were picked and analyzed by 3′ junction PCR for evidence of correct targeting (Figure S9B). To rule out potential false-positives, we performed 5′ junction PCR on genomic DNA from 3′ junction PCR-positive colonies (Figure S9C). A sequence analysis of the resulting 1030 bp (right homology arm) and 1316 bp (left homology arm) fragments of 3′ and 5′ junction PCR confirmed site-specific integration of the targeting vector into the b*ROSA26* locus (Figure 2A). These targeted colonies were continued to culture and passage in vitro. When cultured up to passage 50, the colony was deemed to achieve immortalization.

Simultaneously, we compared immortalization efficiency of the strategy established in our study with traditional immortalization methods, transient transfection or lentivirus-mediated delivery system according to the statistics of the passages. The results showed that the primary BFFs transiently transfected with b*TERT* were cultured for no more than 15 generations. The use of the lentivirus system to mediate the stable integration of b*TERT* to realize cell immortalization was inefficient, and the number of passages of BFFs was increased compared with the transient transfection method, but most did not exceed 50 generations. The proportions of immortalized cells in puromycin-resistant cells in the three independent experiments were 18.18%, 27.27%, and 33.33%. After the accurate integration of b*TERT* mediated by the CRISPR/Cas9 system, BFFs were able to realize immortalization with high efficiency. In three independent experiments, the percentages of immortalized cells in puromycin-resistant cells were 53.85%, 50.00%, and 56.25%. The passage number of immortalized BFFs driven by precise integrated b*TERT* was higher than the two other methods, with a significant proportion even exceeding 80 generations (Table 2). Subsequently, by comparing the relative telomere length and the number of EdU-positive cells, we found that the 15th passage of bTERT-expressing BFF colonies via transient transfection was still close to wild-type 1st passage of BFFs in the two indicators (Figure 2B), and 50th passage of b*TERT*-integrated BFFs via lentivirus-mediated delivery system was close to or even higher than wild-type 1st passage of BFFs (Figure 2C); the 80th passage of b*TERT*-integrated BFFs via the CRISPR/Cas9 system was similar to primary BFFs in both indicators (Figure 2D). In addition, the number of passages, relative telomere length, and number of EdU-positive cells suggested that, regardless of the method of *TERT* introduction, the relative telomere length and the number of EdU-positive cells were higher in BFFs expressing bTERT than in those expressing hTERT with the same strategy (Figure 2B–D and Figure S10). Thus, the immortalization strategy through HMEJ-based CRISPR/Cas9-mediated site-specific b*TERT* insertion at the b*ROSA26* locus established in this study demonstrated an easy operation process and high efficiency. The immortalized cells presented obvious advantages in terms of the number of serial passages and cell viability.

### 2.3. Characteristics of Immortalized Bovine Cells Established via HMEJ-Based bTERT Integration

**Figure 3:**
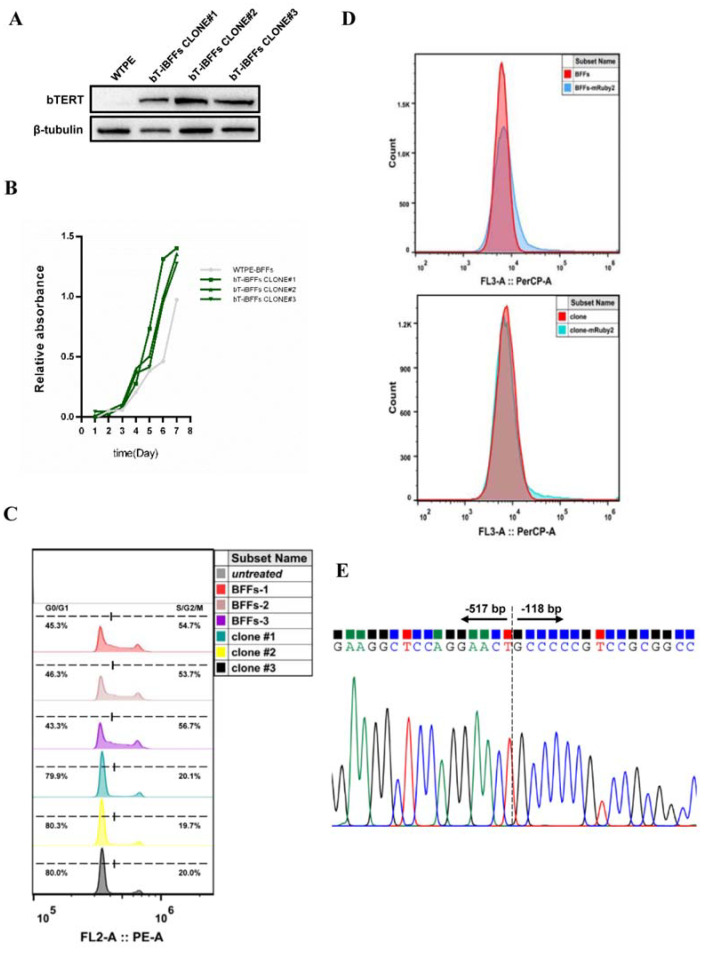
Characteristics of bT₋iBFFs by HMEJ₋based CRISPR/Cas9₋mediated b*TERT* insertion at the b*ROSA26* locus. (**A**) Protein expression of bTERT in bT₋iBFF colonies detected by Western blot. “WTPE” represents wild₋type. Bovine β₋tubulin served as internal reference. (**B**) Cell proliferation abilities of 1st passage of wild-type BFFs and 80th passage of bT₋iBFFs clones by CCK8 cell proliferation assay. (**C**) Cell cycle comparison between 1st passage of primary BFFs and 80th passage of bT₋iBFF colonies. (**D**) Comparison of the gene manipulation efficiency of the primary BFFs and bT₋iBFFs by FACS. Non₋transfected cells were used for negative control. (**E**) Sanger sequencing results of target sites of the b*TERT* promoter region in bT₋iBFFs after the predicted *p53* and *WT1* binding sites were knocked out.

To verify the characteristics and stability of the immortalized BFFs generated by this strategy (referred to as “bT-iBFFs” interchangeably hereafter), we conducted a series of identifications. First, Western blot demonstrated the stable expression of exogenously integrated b*TERT* in bT-iBFFs (Figure 3A). In addition, the expression level of fibroblast marker *VIMENTIN* in bT-iBFFs was similar to primary BFFs (Figure S11A), indicating that the cell type of bT-iBFFs had not changed. Simultaneously, we cloned eight main sgRNA3 potential off-target sites that were predicted based on sequence similarity to the target sequence from the genome of three bT-iBFF colonies and did not detect any typical inserts and/or deletions in the analyzed off-target sites (Figure S12). To test whether the bT-iBFFs underwent malignant transformation, we selected three bT-iBFF colonies that had passaged 80 generations continuously to perform soft agar assay. The results showed that both primary BFFs and bT-iBFFs could not form clones, while Hela cells formed typical cell clones (Figure S11B), indicating that no malignant transformation occurred in the bT-iBFFs. A karyotype analysis of the bT-iBFF colony was performed, showing 60 chromosomes (30 pairs) (Figure S11C), consistent with primary BFFs. The senescence and relative telomere length of bT-iBFFs were examined to reflect cell activity and proliferation potential. A β-Galactosidase staining assay showed that the 80th passage of bT-iBFFs had normal morphology, with only a small amount of β-galactosidase staining, which was similar to the 1st passage of primary BFFs with active proliferative ability (Figure S11E). The relative telomere length assay revealed that passages did not shorten the length of telomere obviously in bT-iBFFs (Figure S11D). A CCK8 cell proliferation curve and cell cycle assay revealed that the proliferation rate of the 80th passage of bT-iBFF colonies was faster than that of primary BFFs (Figure 3B), but the proportion of bT-iBFF colonies in each phase of the cell cycle was different from that of primary cells. The percentage of cells in the G0/G1 phase of bT-iBFFs (79.9%, 80.3%, and 80.0% of three colonies) was higher than that of primary BFFs (45.3%, 46.3%, and 43.3% of three samples), and the proportion of bT-iBFFs in the S/G2/M phase was lower than that of primary BFFs (Figure 3C). Combined with the above results of the EdU staining assay and CCK8 cell proliferation curve, we thought that the sustained high expression level of bTERT accelerated the synthesis of cell DNA and histones associated with chromatin formation, sped the process of the S/G2/M phase, shortened the cell division cycle, and promoted cell proliferation.

The applicability and subsequent transgenic operation of the immortalized cells are usually limited due to the integration of exogenous genes, drug selection, or expand culture during the process of immortalization. Therefore, we explored the transgenic manipulation efficiency of bT-iBFFs. First, we detected the gene operation efficiency via comparing the average fluorescence intensity of bT-iBFFs and primary BFFs by flow cytometry after transfecting pcDNA3-mRuby2 fluorescent protein expression vector. The results revealed a slight difference in the transfection efficiency between primary BFFs and bT-iBFFs; the average fluorescence intensity of primary BFFs or bT-iBFFs transfected with pcDNA3-mRuby2 was 2.35 times or 1.9 times as much as the control group (Figure 3D). The secondary gene operation efficiency of bT-iBFFs did not significantly decrease due to the previous immortalization process. Second, we tested the gene editing efficiency of bT-iBFFs via knockout of the partial promoter region of b*TERT*. By analyzing the binding sites of repression factors *WT1* and *p53* in the 1500 bp promoter region upstream of the b*TERT* transcription start site using PROMO, TFBD, and JASPAR prediction tools (Figure S13A), we selected −600 bp to −60 bp region of the b*TERT* proximal promoter as the targeted region. Thus, we designed two sgRNAs located in −517 and −118 bp of the b*TERT* promoter and then deleted this region in bT-iBFFs and primary BFFs via CRISPR/Cas9 method. Finally, both the BFFs and bT-iBFFs manipulated by the CRISPR/Cas9 knock-out system additionally amplified a band of about 250 bp (Figure S13B), which was identified by Sanger sequencing to be consistent with the genomic sequence after deleting the −517 bp to −118 bp region of the b*TERT* promoter (Figure 3E). These results indicated that the immortalized bT-iBFFs had the same potential for secondary gene editing as primary BFFs.

### 2.4. bTERT-Driven Immortalization via the CRISPR/Cas9 System Inhibited by the Post-Translational Modification in bBMMs

**Figure 4:**
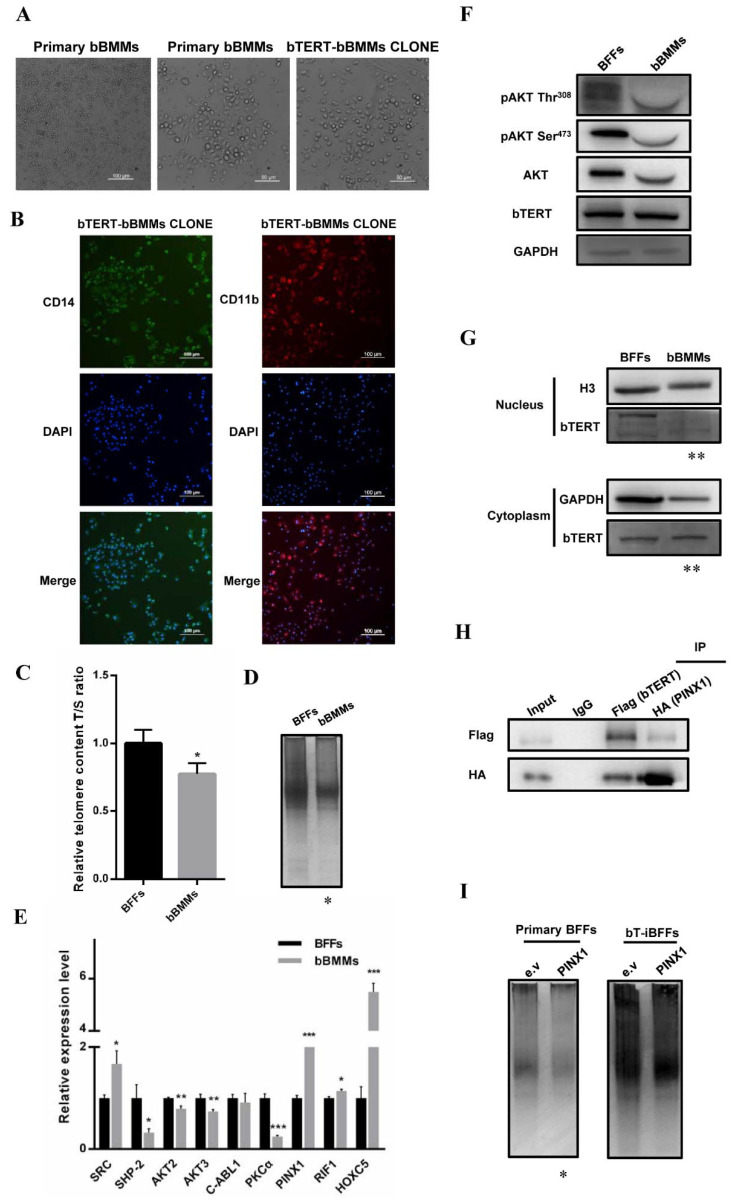
bTERT-driven immortalization via the CRISPR/Cas9 system inhibited by post-translational modification in bBMMs. (**A**) Cellular morphology of bBMMs (scale bar = 100 μm/50 μm) and b*TERT*-bBMM colony (scale bar = 50 μm). (**B**) The expression of macrophage markers CD14 and CD11b in b*TERT*-bBMM colony detected by immunofluorescence. (scale bar = 100 μm). (**C**,**D**) Relative telomere length (**C**) and telomerase activity (**D**) of primary BFFs and bBMMs. Bovine β-globin served as internal reference. (**E**) Endogenous expression of b*TERT*-related post-translational phosphorylation modification modulators in BFFs and bBMMs. Bovine β-actin served as internal reference. (**F**) The protein expression of bTERT, AKT, pAKT Thr^308^, and pAKT Ser^473^ in BFFs and bBMMs. Bovine GAPDH served as internal reference. (**G**) The expression level of bTERT protein in the nucleus and cytoplasm of BFFs and bBMMs. Bovine H3 or GAPDH served as internal reference of nuclear or cytoplasmic proteins, respectively. (**H**) Flag-tagged bTERT and HA-tagged PINX1 were transiently co-expressed in HEK-293T cells. Flag-tagged bTERT or HA-tagged PINX1 was immunoprecipitated, and precipitates were blotted for Flag-tagged bTERT and HA-tagged PINX1. Total cell lysate (input) was set as internal reference for co-immunoprecipitation assay, and normal bovine IgG served as a negative control. (**I**) Telomerase activity of primary BFFs and bT-iBFFs transfected with PINX1 by trap analysis. e.v, empty vector. Data were mean ± s.d., *n* = 3 independent experiments, one-tailed Student’s *t*-test. * *p* < 0.05, ** *p* < 0.01, *** *p* < 0.001 vs. e.v (*I*) or BFFs (**C**–**E**,**G**,**I**).

We then made further extension attempts on other bovine primary cells. Bovine bone marrow-derived macrophages (bBMMs) are critical to the study of bovine cytophagy and cellular and molecular immunity, but the difficulty of the long-term culture of bBMMs greatly limits research on bovine disease. Therefore, we applied the strategy established in the study to immortalize bBMMs and successfully screened cell colonies of bBMMs with b*TERT* integrated (referred to as “bT-bBMMs” interchangeably hereafter). The bT-bBMMs exhibited typical macrophage-like morphology with ruffled membranes, and cell processes resembled primary bBMMs (Figure 4A). Immunofluorescence demonstrated that bT-bBMMs were positive for macrophage markers (*CD11b* and *CD14*) (Figure 4B). Unfortunately, bT-bBMM colonies cultured in vitro were no more than eight passages, and failed to realize immortalization. Although we had obtained bBMMs integrated with b*TERT*, the number of positive clones was only four, three, and three in three independent experiments. By comparing the key factors of the nonhomologous end-joining (NHEJ) repair pathway and homologous recombination (HR) repair pathway [27,28,29], we found that the NHEJ repair pathway played a more important role in bBMMs (Figure S14). Thus, NHEJ-based CRISPR/Cas9-mediated b*TERT* was inserted at the b*ROSA26* locus (Figure S15). Similarly, the number of b*TERT*-integrated bBMMs colonies showed a certain increase with 10, 11, and 11 in three independent experiments, but these colonies still did not achieve immortalization. We ruled out the possibility that the proliferative potential of bBMMs influences the immortalization process, because the proliferation activity, cell cycle, and expression levels of proliferation-, senescence-, and apoptosis-related genes in bBMMs resembled BFFs (Figure S16A,B). Notably, cell cycle results also showed a certain proportion of apoptotic cells in the bBMM cell population (Figure S17C), which may contribute to the difficulties in achieving immortalization. In accordance with the above results and combined with previous studies [19,20,21,22,23,24,25,26,27,28,29,30], b*TERT* cooperated with another immortalization factor *SV40LT* to immortalize bBMMs. Therefore, we inserted b*TERT* and *SV40LT* simultaneously at the b*ROSA26* locus of the bBMM genome via the NHEJ- or HMEJ-based CRISPR/Cas9 system (Figure S17), but still failed to immortalize bBMMs. The above results indicated that the strategy of CRISPR/Cas9-mediated site-specific b*TERT* insertion had certain obstacles in the process of bBMM immortalization, but it was unrelated to the proliferation potential of bBMMs.

The normal function of hTERT in primary cells is necessary for immortalizing human cells [24]. Given that BFFs were immortalized successfully in this study, we used BFFs as a control to investigate the differences in *TERT* and telomerase regulation between primary bBMMs and BFFs. First, we examined the telomerase activity, and the relative telomere length was lower in bBMMs than in BFFs (Figure 4C,D), suggesting that telomerase was inhibited endogenously in bBMMs. QPCR showed that the expression level of *HOXC5*, a post-transcriptional inhibitory factor of *TERT* [31], was significantly higher in bBMMs than in BFFs. However, the expression level of *TERT* and *TERC* was not significantly different (Figure S18). Thus, *bTERT* and b*TERC* were not regulated by an additional repressor at the transcription level in bBMMs. The expression of b*TERT* integrated in the genome was driven by the constitutive promoter CMV using the strategy in this study. The function of exogenous bTERT was possibly regulated by endogenous post-translational regulatory proteins. Post-translational phosphorylation modification is an important factor in regulating the subcellular localization and activity of hTERT [32]. We found that *PKCα*, a positive regulator of intracellular telomerase activity by phosphorylating hTERT [33,34], was significantly lower in bBMMs than in BFFs (Figure 4E). Subsequently, we examined the expression of AKT kinase, promoting hTERT transport from the cytoplasm to the nucleus by phosphorylating hTERT [35,36,37]. The results showed that expression levels of *AKT2* and *AKT3* were significantly lower in bBMMs (Figure 4E), and the protein expression level of total AKT and activated phosphorylation modification levels of Thr^308^ and Ser^473^ were also low in bBMMs (Figure 4F). In bBMMs, the expression level of cytoplasmic transport promoting factor *SRC* kinase [38] was higher, whereas phosphatase *PTPN11* [39], as opposed to *SRC*, was lower (Figure 4E), thereby suggesting that the nuclear translocation of TERT was inhibited in bBMMs. The results of the similarity in the bTERT expression level of bBMMs and BFFs (Figure 4F) indicated that the discrepancy in telomerase function in BFFs and bBMMs was not caused by the different expression level of TERT, pointing to the difference in the subcellular localization of TERT. To confirm our hypothesis, we examined protein levels of nuclear and cytoplasmic bTERT in bBMMs and BFFs, respectively. The results showed that the protein level of nuclear bTERT in BFFs was higher than that in bBMMs; by contrast, the protein level of cytoplasmic bTERT in bBMMs was higher than that in BFFs (Figure 4G). This result demonstrated that the localization of TERT in the two primary cells greatly differed, with predominant nuclear localization in BFFs, but a tendency of cytoplasmic distribution in bBMMs, which probably prevented the normal function of endogenous and exogenous bTERT in bBMMs. Previous studies have demonstrated that PINX1 can isolate TERT in the nucleolus and negatively regulate telomerase activity in human cells [40,41,42]. The QPCR experiment showed that the expression level of *PINX1* was significantly higher in bBMMs than in BFFs (Figure 4E), and Co-IP experiment also revealed the protein interaction between PINX1 and bTERT (Figure 4H), which was consistent with hTERT. An overexpression of PINX1 in BFFs resulted in a notable decrease in telomerase activity; simultaneously, an overexpression of PINX1 in bT-iBFFs inhibited telomerase activity (Figure 4I). The above results implied that the high expression of PINX1 inhibited the function of bTERT by interacting with bTERT, thereby inhibiting telomerase activity. In addition, we compared the expression levels of the components of the “Shelterin” complex and CST complex, which play crucial roles in preventing unregulated telomere elongation [43,44] and limiting the endogenous activity of TERT and telomerase in bBMMs and BFFs [45], respectively. QPCR experiments revealed that both the “Shelterin” complex and CST complex were active in bBMMs (Figure S19), and may synergistically inhibit telomerase binding to chromosome ends.

## 3. Discussion

In this study, a novel immortalization strategy with easy manipulation, secure applicability, and high efficiency for bovine primary cells was established via a HMEJ-based CRISPR/Cas9-mediated site-specific b*TERT* insertion at the b*ROSA26* locus. We chose to use bovine *TERT* to immortalize BFFs not only to avoid potential risks caused by the introduction of heterogeneous genes from other species such as h*TERT*, but to maximize the advantages of b*TERT* over h*TERT* for the immortalization of bovine cells. A previous study found that h*TERT* can be used for the immortalization in cells of various species. The CTD of hTERT is critical in the process of immortalization, but mTert has defects in achieving immortalization [24,25]. This result suggests obvious discrepancy in the function of TERTs from different species. A comparison of the effects of bTERT, hTERT and mTERT on the telomerase activity and telomere length revealed that bTERT was more advantageous than the others in enhancing telomerase activity and maintaining telomere length. Moreover, the CTD of bTERT was more active than those of hTERT and mTERT. The dominance of bTERT was particularly prominent in the primary cells of livestock, such as cattle and goat.

In previous reports, immortalized cell lines were usually constructed through introducing immortalization factors via transient transfection or virus-mediated delivery system, but both methods have drawbacks. The method of transient transfection is unable to maintain the stable expression of exogenous immortalization factors, which greatly reduces the efficiency of primary cells to achieve immortalization. Although the method of virus-mediated delivery system overcomes the defect of the short expression time of exogenous immortalization factors, this method causes great damage to the genome due to random exogenous gene integration and unknown copy numbers. To overcome the shortcomings of the two methods, we chose to integrate b*TERT* into the b*ROSA26* locus precisely via the CRISPR/Cas9 system in order to establish immortalized cell lines. We then compared the efficiency of this strategy with other traditional immortalized methods in bovine cell immortalization. By counting the number of immortalized cell lines by these different strategies, we revealed that the efficiency of the CRISPR/Cas9 system was highest for generating immortalized bovine cell lines, and b*TERT* was more advantageous for bovine cell immortalization than h*TERT*. In addition, the characteristics, proliferation activity and secondary gene editing efficiency of the immortalized BFFs driven by the CRISPR/Cas9-mediated integration of b*TERT* resembled primary BFFs, proving that the strategy was efficient, reliable, and practical.

Current immortalization methods generally have their own applicable cell types, so they are often limited in practical application. We found that the immortalization strategy established in this study was applicable to BFFs, but had some limitations for bBMMs immortalization. In this study, we found that bTERT was controlled at the post-translational and subcellular levels in bBMMs. Previous studies revealed that kinase-mediated phosphorylation affects the subcellular localization of hTERT and limits the function of telomerase [32]. As a multifunctional kinase, AKT kinase phosphorylates and regulates hTERT in various ways. hTERT Ser^227^ phosphorylation by AKT increases the binding affinity of hTERT with the nuclear import receptor importin-α, which promotes the nuclear import of hTERT, thereby increasing telomerase activity [35,36,37]. In bBMMs, we found that the expression level of AKT and activated phosphorylation modifications Thr^308^ and Ser^473^ was lower than in BFFs, implying that the low activity of AKT kinase restricted the translocation of bTERT to the nucleus and became trapped in the cytoplasm. The more direct evidence was that the protein level of nuclear bTERT was significantly higher in BFFs than in bBMMs, while the protein level of cytoplasmic bTERT was obviously higher in bBMMs. According to Liu et al. [46], during human CD4^+^ T lymphocyte activation, CD4^+^ T cells regulate telomerase function independent of the hTERT protein level, but they are involved in the phosphorylation and nuclear translocation of hTERT. Therefore, the function of endogenous telomerase in bBMMs is regulated by the phosphorylation and cytoplasmic transport of bTERT. In addition, other telomere-associated proteins and complexes participate in the inhibition of TERT. In human cells, PINX1 inhibits telomerase function by forming a stable complex with the catalytic subunit of telomerase and TRF1 of the “Shelterin” complex and binding to TERT through its C-terminal telomerase inhibitory domain containing 74 amino acids, which leads to the isolation of TERT in the nucleolus and negative regulation of telomerase activity [40,41,42]. Interestingly, we found that *PINX1* was expressed higher in bBMMs than in BFFs and interacted with bTERT to inhibit the function of bTERT, thereby restricting telomerase activity. In addition, other telomere-related protein complexes regulate telomerase function. Studies have reported that the “Shelterin” complex binds to the G-overhang structure of telomere, promotes the formation of T-loop, hides the 3’ end of telomeric DNA, blocks telomerase from binding to chromosome ends, and prevents unregulated telomere elongation [43,44]. The CST complex, another telomerase-related complex in mammalian cells, directly binds to the nascent G-overhang structure and prevents telomerase-mediated telomere elongation [45]. In this study, we found that the activity levels of the “Shelterin” complex and CST complex were remarkably higher in bBMMs than in BFFs, contributing to the low telomerase activity and short telomere length of bBMMs. Therefore, in designing strategies for primary bBMMs immortalization, we could superimpose the gene editing system to introduce b*TERT* and eliminate negative regulators of b*TERT* simultaneously, ensuring the normal function of exogenous bTERT in bBMMs, maintaining intracellular telomerase activity and telomere length, and immortalizing bBMMs.

The greatest significance of this study is the establishment of a novel strategy for immortalizing bovine primary cells, which has obvious advantages over traditional methods. By implanting the CRISPR/Cas9 gene editing method into the immortalization strategy, we did not only overcome the short duration of immortalization factor expression, but also avoided the potential disruption of genomic stability by the random integration of exogenous genes. Meanwhile, using bovine *TERT* to achieve the immortalization of bovine primary cells was significantly more efficient than using human *TERT*, suggesting that the suitability of different species of *TERT* must be considered during immortalization. In addition, certain factors limit the function of TERT in terminally differentiated cells, such as macrophages. Therefore, the expression and regulatory patterns of TERT should be considered in future immortalization strategies, and protocols should be customized on this basis to eliminate endogenous factors that inhibit TERT function, resulting in new perspectives of cell immortalization.

## 4. Materials and Methods

### 4.1. Cell Isolation and Culture

Primary BFFs were isolated from 35~40-day–old fetuses. The tissues were minced, plated on 60-mm Petri dishes (Corning Costar, #430166, USA), and cultured with DMEM/F12 (Gibco, #10565018, NY, USA) supplemented with 10% fetal bovine serum (FBS) (Gibco, #10100-147, Australian) at 37 °C in an atmosphere of 5% CO_2_. The cells were harvested using 0.25% trypsin/EDTA solution (Gibco, #25200072, NY, USA) and frozen in 90% FBS and 10% DMSO (Sigma-Aldrich, #D2650, MO, USA). BFFs were thawed and grown in a DMEM/F12 medium with 10% FBS, and incubated at 37 °C in a 5% CO_2_ environment when needed. The isolation of primary MEFs and GFFs was performed as primary BFFs.

Primary bBMMs were isolated from the bones of 90-day-old fetuses. The following describes the procedure. First, detach the femurs from fetuses and remove residual tissues. Second, cut the epiphysis of each femurs. Third, flush each bone marrow cavity with RPMI 1640 (Gibco, #31800-022, St Louis, MO, USA) using a 10 mL syringe until the bone appears mostly white. Bone marrow cells were harvested and cultured in the RPMI 1640 containing 10% FBS (Gibco, #10100-147) and 25 ng/mL GM-CSF (Sigma-Aldrich, #SRP3201) at 37 °C in a 5% CO_2_ environment. After 7-days induction, the non-adherent cells were removed and the remaining adherent cells were used for subsequent experiments.

HEK-293T cells (ATCC, VA, USA) and Hela cells (ATCC, VA, USA) were cultured with DMEM (Gibco, #12800-082, Waltham, MA, USA) supplemented with 10% FBS (Gibco, #10100-147).

### 4.2. Construction of Vectors

The full-length coding sequence of mouse Tert was amplified from mouse ovary cDNA and subcloned into p3×Flag-CMV-10 and pC1-neo. The full-length coding sequence of bovine *TERT* was also amplified from bovine ovary cDNA and subcloned into p3×Flag-CMV-10, pC1-neo, and pLenti-puro. The full-length coding sequence of human *TERT* was provided by Y.P. Jin (College of Veterinary Medicine, Northwest A&F University) and subcloned into p3×Flag-CMV-10 and pLenti-puro. C-terminal deletion mutants of mTert, b*TERT*, and h*TERT* were generated by standard PCR methods. Other C-terminal substitute recombinant vectors of mTert, b*TERT*, and h*TERT* were spliced using a pEASY^®^-Basic Seamless Cloning and Assembly Kit (Transgene, #CU201, Beijing, China), as shown in Figure 1A. The full-length coding sequence of SV40LT was amplified from HEK-293T cell cDNA. The full-length coding sequence of *PinX1* was amplified from BBFs and subcloned into pCMV-HA. The primers used to clone these plasmids are available in Table S1. The full-length sequence of PINX1 was amplified from bovine ovary cDNA and subcloned into pCMV-HA, and the primers are available in Table S1.

Cas9/sgRNA1-puro, Cas9/sgRNA2-puro, Cas9/sgRNA3-puro, Cas9/sgRNA4-puro, and Ca9/sgRNA8 were generated based on pSpCas9(BB)-2A-Puro (PX458, Addgene plasmid #48139). The pCMV-b*TERT*-pEF1α-EGFP-PURO-HMEJ gene-targeting vector was constructed as shown in Figure S9A. The vector contains a pair of sgRNA3, and a 5′ arm and a 3′ arm of homology, which together span 1623 bp of the b*ROSA26* locus. The vector overlaps with sequences of the exon 1 and the intron 1of the b*ROSA26* locus. The selected markers cassette consists of the EGFP and puromycin resistance gene, which were fused by the porcine teschovirus-1 2A peptide sequence. The transcription of the selected markers was driven by an EF1α promoter. The LoxP sites were positioned such that after the expression of Cre recombinase (Cre), the selected markers cassette was removed after subsequent exogenous gene target for the production of marker-free gene-edited immortalized cells. CMV promoter with b*TERT* was inserted into pEF1α-EGFP-PURO-HMEJ vector, directing b*TERT* expression in cells stably. The pCMV-b*TERT*-pEF1α-EGFP-PURO-NHEJ gene-targeting vector was constructed by removing the 5′ arm and 3′ arm of the pCMV-b*TERT*-pEF1α-EGFP-PURO-HMEJ according to the Figure S15. The pCMV-b*TERT*-IRES-*SV40LT*-pEF1α-EGFP-PURO-NHEJ gene-targeting vector was constructed by inserting IRES with *SV40LT* after b*TERT* of the pCMV-b*TERT*-pEF1α-EGFP-PURO-NHEJ as shown in Figure S17. The pCMV-b*TERT*-IRES-*SV40LT*-pEF1α-EGFP-PURO-HMEJ gene-targeting vector was constructed by inserting IRES with *SV40LT* after b*TERT* of the pCMV-b*TERT*-pEF1α-EGFP-PURO-HMEJ as shown in Figure S17. The primers used to clone these plasmids are available in Table S1.

### 4.3. Cell Transfection

HEK-293T cells, Hela cells, and primary bBMMs with a confluence of 70% to 90% were used for transfection according to the protocol of the Lipofectamine2000 reagent and Lipofectamine3000 reagent (Thermo Fisher, #L3000-150).

BFFs, MEFs, and GFFs were thawed and grown in DMEM/F12 medium supplement with 10% FBS, and incubated at 37 °C in a 5% CO_2_ environment before electroporation. At 70–80% confluency, cells were trypsinized and resuspended in Opti-MEM (Gibco, #31985062, NY, USA) with 10 μg plasmid, and electroporated at 510 V with two pluses of 2-ms duration using the BTX Electro-cell manipulator ECM2001 (BTX Technologies, CA, USA). The electroporated cells were plated on cell dishes at a suitable density. The cells used for selecting individual colonies were screened and expanded after puromycin selection 12–14 days (1 μg/mL 12~13 after 1.5 μg/mL 1~2 days) after electroporation. The cells used in other experiments were cultured for a suitable time after electroporation.

Lentivirus plasmids were transfected into HEK-293T cells together with packaging and envelope plasmids (psPAX2 and pMD2.G) using Lipofectamine2000 (Thermo Fisher, #11668019). At 2 days after transfection, the medium was centrifuged at 14,000× *g* at 4 °C for 15 min and then passed through a 0.45 μm pore filter. The medium containing lentiviruses was transferred to primary BFFs. HEK-293T cells were further cultured in a fresh medium for 24 h. After 24 h of infection, medium was changed, and cells were cultured in a DMEM/F12 medium with 2 μg/mL DOX (Sigma-Aldrich, #D3072) and selected with 1 μg/mL of puromycin for 8~10 days.

### 4.4. Reverse Transcription PCR and Quantitative RT-PCR

Total RNA was isolated from cells, along with various adult tissues using the RNAiso reagent (Takara, #9109, Dlian, China) according to the manufacturer’s instruction. Purified RNA was reverse-transcribed using a HiScriptⅡ1st Strand cDNA Synthesis Kit (+gDNA wiper) (Vazyme Biotech, Nanjing, China). A quantitative real-time polymerase chain reaction (qPCR) was performed in a technical triplicate using SYBR Premix ExTaq II (Takara, #RR820A) with an ABI StepOnePlus PCR system (Applied Biosystem, CA, USA). All data were generated using cDNA from triplicate wells for each condition. The comparative Ct method was used to calculate the relative quantity of the target gene mRNA, normalized to bovine β-actin, and was expressed as the fold change =2^−∆∆Ct^. The following procedures were used for qPCR experiments: 30 s at 95 °C, followed by 40 cycles of 5 s at 95 °C, and 30 s at 60 °C. The primer sequences used for qPCR are available in Table S1.

### 4.5. Western Blot

Cell or liquid nitrogen grinded tissues were lysed in ice-cold RIPA cell buffer supplemented with a protease inhibitor (Thermo Scientific, NH, USA) or phosphatase inhibitor (Thermo Scientific, NH, USA). The proteins were fractionated by sodium dodecyl sulphate (SDS)-polyacrylamide gel electrophoresis and transferred to polyvinylidene difluoride membranes (Millipore, #ISEQ00010, Burlington, MA, USA). The membranes were blocked in 5% non-fat milk powder in Tris-buffered saline with 0.1% Tween-20 (TBST) and subsequently incubated with the primary antibody at 4 °C overnight. The membranes were incubated with horseradish peroxidase (HRP)-conjugated secondary antibody at room temperature the next day. After being washed with TBST, the membranes were revealed by autograph using WesternBright ECL kit (Advansta, #K-12045-D50, Menlo Park, CA, USA). The antibodies used (at 1:1000 unless otherwise noted) were anti-TERT (1:800, Abcam, #ab32020, UK), anti-HA (Beyotime, #AF0039, Shanghai, China), anti-histone H3 (1:500, Proteintech, #17168-1-AP, Shanghai, China), anti-AKT (Cell Signaling Technology, #92725, Shanghai, China), anti-pAKT Thr^308^ (Cell Signaling Technology, #13038), anti- pAKT Ser^473^ (Cell Signaling Technology, #4060), anti-glyceraldehyde-3-phosphate dehydrogenase (GAPDH) (1:2000, Beyotime, #AF0006), and anti-β-tubulin (1:2000, Transgene, #HC101).

### 4.6. Establishment of Immortalized Bovine BFFs and bBMMs Using the HMEJ-Based CRISPR/Cas9-Mediated bTERT Expression at ROSA26 Locus

Early passage BFFs (<3 passages) were seeded in 60 mm^2^ dishes and co-transfected with 5 μg of pCMV-b*TERT*-pEF1α-EGFP-PURO-HMEJ and 5 μg Cas9/sgRNA3 by electroporation. At 48 h after transfection, cells were subjected to puromycin selection. A stable cell pool was obtained and continuously passaged for further analysis.

Primary bBMMs that were induced by GM-CSF for 7 days were seeded in 60 mm^2^ dishes and co-transfected with 3 μg of donor plasmid and 3 μg Cas9/sgRNA3 by Lipofectamine3000. At 48 h after transfection, cells were subjected to puromycin selection. A stable pool was obtained, and cultured in fresh RPMI 1640 containing 10% FBS and decreasing the concentration of GM-CSF. There was a gradual decrease in the concentration of GM-CSF until bBMMs are able to survive and proliferate in the absence of any GM-CSF conditioned media.

### 4.7. RNA Binding Protein Immunoprecipitation Assay

Before the cells were collected, the Pierce Protein A/G Agarose were coated with FLAG mAb (Sigma Aldrich, #F1804, Saint Louis, MO, USA) or control immunoglobulin G at 4 °C for 6 h. The 1 × 10^7^ cells were trypsinized and resuspended in ice-cold PBS, followed by cross-linked by 37% formaldehyde for 15 min. Subsequently, 2 m glycine was added to neutralize the formaldehyde. Cells were centrifuged at 100× *g* for 2 min, and washed with ice-cold PBS twice. Cell extracts were lysed in RIP lysis buffer (25 mM Tris-HCl of pH 7.5, 150 mM KCL, 2 mM EDTA, 0.5% NP40, 1 mM NaF, 1 mM DTT, 100 U/mL RNase inhibitor, 0.1 mM PMSF) for 30 min on ice and clarified via centrifuged. Protein extracts were incubated with pre-coated Pierce Protein A/G Agarose at 4 °C overnight, and washed twice in an ice-cold low salt buffer (50 mM Tris-HCl of pH7.4, 150 mM NaCl, 1 mM MgCl_2_, 0.05% NP40, 2 mM EDTA, 1 mM DTT, 100 U/mL RNase inhibitor) and once in an ice-cold high salt buffer (50 mM Tris-HCl of pH 7.4, 300 mM NaCl, 1 mM MgCl_2_, 0.05% NP40, 2 mM EDTA, 1 mM DTT, 100 U/mL RNase inhibitor). Then, RIP buffer (50 mM Hepes of pH 7.5, 0.1 M NaCl, 5 mM EDTA, 10 mM DTT, 0.1% TritonX-100, 10% Glycerol, 1% SDS, 100 U/mL RNase inhibitor) was added to the beads, and incubated at 70 °C for 1 h to remove the crosslinking. The beads were precipitated by centrifugation at 400× *g* for 1 min at room temperature, and collected the supernatant. The supernatant was added with RNAiso reagent, and then extracted the RNA to perform quantitative RT-PCR. Primer sequences for quantitative RT-PCR are listed in Table S1.

### 4.8. Telomerase Activity Assay

Cells were harvested and washed in PBS, and then lysed in ice-cold CHAPS Lysis Buffer (0.5% CHAPS, 10 mM Tris-HCl of pH 7.5, 1mM MgCl_2_, 1 mM ethylene glycol-bis (β-aminoethyl ether)-N,N,N’,N’-tetraacetic acid (EGTA), 5 mM β-mercaptoethanol, 10% glycerol, 0.1 mM phenylmethanesulfonyl fluoride (PMSF), 200 units/mL RNase inhibitor). During this period, cells were vortexed every 5 min to prevent subsidence. After being incubated on ice for 30 min, the lysate was centrifuged at 16,000× *g* for 30 min at 4 °C to remove debris. The protein concentration of each sample was measured by an Epoch MultiVolume Spectrophotometer System (BioTek, USA), and 10 μg of total protein was analyzed by TRAP silver stain according to previous studies [47]. The primers sequences are listed in Table S1. Briefly, each reaction contained 5 μL 10×TRAP buffer (200 mM Tris-HCl of pH8.3), 1.5 mM MgCl_2_, 630 mM KCl, 0.05% Tween-20, 1.0 mM EGTA, 1 mg/L BSA), 0.125 μL 10 mM dNTPs, 0.1 ng TS primer, 0.25 μL rTaq (Takara, #RR001A), 10 μg protein concentration, and was adjusted to 50 μL of total volume with RNase-free water. The assay procedure comprised 30-min of incubation at 25 °C, followed by 2-min incubation at 94 °C, and 2-min incubation at 4 °C. Subsequently, 0.1 ng of CX primer was added into this volume, and proceeded with a new reaction. The conditions were 32 cycles of 30 s at 94 °C, 30 s at 55 °C, and 90 s at 72 °C, followed by 10 min at 72 °C. The products obtained in this step were resolved by electrophoresis using a nondenaturing 10% (*w/v*) polyacrylamide gel electrophoresis (PAGE) in a buffer containing 54 mM Tris-HCl (pH 8.0), 54 mM boric acid, and 1.2 mM EDTA. The gel was stained with silver and then photographed under the Alliance Q9 Advanced Gel Documentation System.

### 4.9. Relative Telomere Length Measurement by Quantitative Real-Time PCR

Telomere qPCR was performed as described in previous studies [48,49] with the following modifications. Genomic DNA was extracted from cells according to the manufacturer’s protocol using the TIANamp Genomic DNA Kit (TIANGEN BIOTECH, #DP304-03, Beijing, China). Primers used to amplify the telomere and the single copy reference gene β-globin for each sample are listed in Table S1. According to our results, 20 μL of the final volume per reaction contained 10 μL of 2× SYBR Premix ExTaq (Takara, #RR820A), 0.8 μL of each telomere primer (Tel1, 90 nM, Tel2, 300 nM) or 0.8 μL of each β-globin primer (150 nM), 5 μL of template DNA (7 ng/μL), and 4.4 μL double-distilled water. The Telomere and β-globin real-time amplifications were performed in the ABI StepOnePlus PCR system (Applied Biosystem, CA, USA). The Telomere qPCR conditions were 10 s at 95 °C, followed by 30 cycles of 5 s at 95 °C, 30 s at 54 °C, and 31 s at 72 °C. The relative telomere length was determined by the T/S ratio of the telomere product amplification (T), to the internal single copy reference gene β-globin (S).

### 4.10. EdU(5′-ethynyl-2′-deoxyuridine) Incorporation Assay

Cells were seeded in 24-well plates overnight, and then detected following the instruction of the BeyoClick^TM^ EdU Cell Proliferation Kit with Alexa Fluor488 (Beyotime, #C0071) the next day, and finally photographed under an inverted fluorescence microscope (Nikon, Tokyo, Japan).

### 4.11. Detection of Individual Colonies by PCR

Puromycin-resistant cell colonies derived from the transfected cell populations were collected by trypsinization, and 80% of these were plated in serum-containing culture medium and expanded. The remaining colonies were resuspended in 20 μL of PCR-compatible lysis buffer (0.9% NP-40; 0.9% Triton X-100; 40 mM Tris-HCL; 0.4 mg/mL proteinase K) for PCR analysis. The lysates were incubated at 65 °C for 15 min and then at 95 °C for 10 min. According to our results, 20 μL of the final volume per reaction contained 5 μL of the DNA lysate, 1 μL of each PCR primers for 3′ junction PCR, and 10 μL of EmeraldAmp (Takara, #RR330A, Shiga, Japan). The PCR was performed under standard conditions. Subsequently, 5′ junction PCR was performed on the positive colonies to confirm the correct targeting events. The primers used for junction PCR are shown as Tables S1 and S2.

### 4.12. Off-Target Analyses

Potential off-target sites in the bovine genome were identified using the Cas-OFFinder (http://www.rgenome.net/cas-offinder/, accessed on 20 June 2018), and eight sites with the highest risk of being edited were selected to be examined. PCR products were obtained by amplification from bT-iBFF colonies genome and performed with Sanger sequencing. The sequences of sgRNA3 potential off target sites were shown at Table S3. The primers were shown at Table S4.

### 4.13. Soft Agar Assay for Colony Formation

For this assay, two different concentrations of agarose gel were prepared in the beginning. The procedure should proceed as follows. Mix 1% sterile agarose gel with antibiotics and equal volumes of 2×DMEM/F12 with 20% FBS was used as the base glue. Add 3 mL of the base glue to each 60 mm petri dish and set aside for 5 min to allow agar to solidify. Then, mix 0.7% sterile agarose gel with 2×DMEM/F12 with 20% FBS to use as the top agarose. Adherent cells are trypsinized and counted. The cell suspension concentration is adjusted to 200,000 cell/mL. Mix 0.1 mL of cell suspension with top agarose to be added to the 60 mm petri dish pre-coated with the base glue. Cells are cultured in soft agar medium at 37 °C for two weeks. Over this period, cells are fed 1~2 times every week with DMEM/F12. Plates are stained with 0.005% crystal violet (Beyotime, #C0121) for more than 1 h and then photographed under an inverted microscope (Nikon, Tokyo, Japan).

### 4.14. Karyotype Analysis

Chromosomes were prepared from bT-iBFFs at passage 80. Cells were exposed to 0.4 μg/mL colchicine (Beyotime, #ST1173) in fresh medium and incubated at 37 °C. After 5 h, cells were trypsinized and collected by centrifugation at 1000 rpm for 5 min. Cells were treated with 0.075 mol/L KCl in a 37 °C water bath for 30 min, and then fixed at the room temperature. The cell suspension was added to the pre-cooled slide, and the slides were stained with Giemsa solution in PBS for 10 min at room temperature.

### 4.15. Senescence-Associated β-Galactosidase Staining

The cell senescence assay was carried out using a Senescence β-galactosidase staining Kit (Beyotime, #C0602) following the manufacturer’s instruction.

### 4.16. Cellular Proliferation Assay

A cell proliferation assay was performed with the Cell Counting Kit (Zeta, #K009, Shanghai, China) in accordance with the manufacturer’s instruction.

### 4.17. Cell Cycle Analysis

A cell cycle analysis was conducted to compare the proliferative ability of primary BFFs, bT-iBFFs, and primary bBMMs. The cells were harvested and fixed with 70% ice-cold ethanol for 1 h at 4 °C, centrifuged at 1000 rpm for 5 min, and then resuspended in ice-cold PBS. Subsequently, the cells were stained with 50 μg/mL propidium iodide (Thermo Fisher, #BMS500PI), 0.2% Triton X-100, 100 μg/mL RNase A (Transgene, #GE101-01) for 30 min in the dark at room temperature. The cell cycle analysis was conducted using an Accuri C6 cell sorter (BD Biosciences, San Jose, CA, USA).

### 4.18. FACS Analyses

To determine the percentage of cells with mRuby2-positive, BFFs and bT-iBFFs were transfected with 8 μg of pcDNA3-mRuby2 via electroporation. Two days after transfection, cells were sorted to purify mRuby2-positive cells using the Accuri C6 cell sorter (BD Biosciences, San Jose, CA, USA) and analyzed with FlowJo data analysis software.

### 4.19. Immunofluorescence Staining

The experiment was carried out according to the Immunofluorescence Staining Kit (Beyotime, #FD008), and then photographed under an inverted fluorescence microscope (Nikon, Tokyo, Japan). The antibodies used were anti-CD11b (1:300, BIO-RAD, #MCA1425, US) and anti-CD14 (1:300, BIO-RAD, #MCA6085, US).

### 4.20. Preparation of Cell Lysates

The cell lysates were extracted by the Nuclear and Cytoplasmic Protein Extraction Kit (Transgene, #P0028), and then analyzed by Western blot.

### 4.21. Co-Immunoprecipitation

Whole-cell extracts were collected via the lysis of 1 × 10^7^ cells in 500 μL 1× IP Lysis Buffer for 30 min on ice and clarified by centrifugation. Protein extracts were incubated at 4 °C overnight with FLAG mAb (Sigma Aldrich, #F1804) or control immunoglobulin G, and precipitated proteins were captured with Pierce Protein A/G Agarose for 6 h at 4 °C. After twice washing in IP Lysis Buffer with a complete protease inhibitor mixture, bound proteins were eluted in 5 × SDS loading buffer and analyzed by Western blot.

### 4.22. Statistical Analysis

*GraphPad PRISM 7* software was used for statistical analyses. Error bars, *p* values, and statistical tests were reported in the figure legends. Statistical tests included unpaired one-tailed or two-tailed Student’s *t*-test and a one-way analysis of variance. *p*-value > 0.05 was considered as not significant (ns), 0.01 < *p* < 0.05 as significant and indicated with one asterisks *, 0.001 < *p* < 0.01 very significant and indicated with two asterisks **, and 0.0001 < *p* < 0.001 extremely significant and indicated with three asterisks ***.

## 5. Conclusions

Here, we established an advanced strategy to generate bovine immortalized cell lines efficiently and stably by inserting a novel potent immortalization factor b*TERT* at the b*ROSA26* locus via HMEJ-based CRISPR/Cas9 system. We then constructed immortalized BFF cell lines with stable characteristics. During the course of extending this strategy, we revealed that the nuclear translocation and activity of endogenous TERT were depressed by the post-translational modifications in bBMMs, which hindered the immortalization process, and contributed to the subsequent immortalization of bovine terminally differentiated cells.

## Figures and Tables

**Table 1:** Summary of activities of *TERT* mutants. Enzymatic activity was determined by TRAP. Telomere length was determined by quantitative real-time PCR. +, telomere maintenance; −, telomere shortened.

Construct	Enzymatic Activity	Telomere Length
bTERT	++++	++++
hTERT	+++	+++
mTERT	−	−
bTERT-△C	++	−
hTERT-△C	−	−
mTERT-△C	−	−
HHB	+++	+++
MMB	++	+
BBH	+	−
MMH	+	−
BBM	−	−
HHM	−	−

**Table 2:** Statistics of the number of BFFs colonies generated by the introduction of h*TERT* or b*TERT* via transient transfection, lentivirus-mediated delivery, and HMEJ-based CRISPR/Cas9-mediated integration, which cultured no more than 0 generation, 5 generations, 15 generations, 35 generations, 50 generations, and more than 80 generations.

Immortalization Strategy		Passage 0	Passage 5	Passage 15	Passage 35	Passage 50	Passage 80
Transient transfection system	hTERT	26	1	0	0	0	0
		25	0	0	0	0	0
		16	0	0	0	0	0
	bTERT	25	2	1	0	0	0
		21	1	3	0	0	0
		30	3	2	0	0	0
Lentivirus- mediated delivery system	hTERT	6	2	1	1	0	0
		9	1	2	1	1	0
		9	3	1	o	2	0
	bTERT	1	3	2	4	1	1
		3	3	4	1	3	0
		4	2	0	2	2	2
CRISPR/Cas9 system	hTERT	2	4	3	1	2	1
		3	2	1	2	1	2
		2	2	3	4	3	1
	bTERT	0	1	2	3	3	4
		1	0	1	3	2	3
		0	0	3	4	2	7

## Data Availability

All data generated or analyzed during this study are included in this article, and the supporting information, tables, or from the corresponding author upon request.

## Supplementary Materials

The following are available online at https://www.mdpi.com/article/10.3390/ijms222212540/s1.

## Abbreviations

HMEJ, homology-mediated end joining; *TERT*, telomerase reverse transcriptase; BFFs, bovine fetal fibroblasts; bBMMs, bovine bone marrow-derived macrophages; qPCR, quantitative real-time PCR; RIP, RNA binding protein immunoprecipitation assay; MEFs, mouse embryonic fibroblasts; GFFs, goat fetal fibroblasts; CTD, the C-terminal extension analogous to a polymerase thumb domain; NHEJ, non-homologous end-joining; HR, homologous recombination; TSS, transcription start site; GAPDH, glyceraldehyde 3-Phosphate dehydrogenase; *p*, *p*-value.
