# Supplementary material for: Establishment of an Efficient Immortalization Strategy Using HMEJ-Based bTERT Insertion for Bovine Cells
Source: Int J Mol Sci. 2021 Nov 21;22(22):12540. doi: 10.3390/ijms222212540 (PMC8622252; doi:10.3390/ijms222212540)
Supplement: Supplementary file 1 [file ijms-22-12540-s001.zip › ijms-1453812-supplementary.pdf]

Supplementary Figures and Tables

Establishment of an efficient immortalization strategy using HMEJ-based b*TERT* insertion for bovine cells

Zihan Zhang [1,2,†], Zhuo Han [1,2,†], Ying Guo [1,2,†], Xin Liu [1,2], Yuanpeng Gao [1,2,*] and Yong Zhang [1,2,*]

Table S1. Primers used for quantitative real-time PCR, RNA binding immunoprecipitation assay, amplification of *TERT*, *SV40LT*, *PINX1* fragment, telomerase activity assay, the relative telomere length measurement, sgRNA cloning and amplification of knock-out target sites.

Name	Primer sequence	Purpose of primer
TERT	Forward primer (5′- 3′): GCTCACGGGTCAAGACGCTGTT	Primers for qPCR
	Reverse primer (5′- 3′): CGGCTGGAAGTCCGAGAAGG	
β-actin	Forward primer (5′- 3′): CGGATGTCGACGTCACACTTCATGA	
	Reverse primer (5′- 3′): ATCACCATCGGCAATGAGCGGTTC	
HoxB8	Forward primer (5′- 3′): ACACAGCTCTTCCCCTGGAT	
	Reverse primer (5′- 3′): AGTCCCAGTGCATGCGATAC	
SIRT1	Forward primer (5′- 3′): GGCTTACAGGGCCTATCCAG	
	Reverse primer (5′- 3′): ACACGAATGGAAACCGTTGG	
BCL2	Forward primer (5′- 3′): TGGCCTTCTTTGAGTTCGGA	
	Reverse primer (5′- 3′): AAATCAAACAGGGGCCGCAT	
JUN	Forward primer (5′- 3′): ACGACCTTCTACGACGATGC	
	Reverse primer (5′- 3′): CCCGTTGCTGGACTGTATGA	
c-Myc	Forward primer (5′- 3′): TACAACATCCGAGCGACACC	
	Reverse primer (5′- 3′): GCAAGCCCGTATTTCCACTATC	
CDK4	Forward primer (5′- 3′): GGCGAGGGTCTTCTCTGGT	
	Reverse primer (5′- 3′): CCTCCTCCATTGGGGACTCT	
FOS	Forward primer (5′- 3′): TTCACCTACCCTGAGGCTGA	
	Reverse primer (5′- 3′): CTCCTCTCTGTAATGCGCCA	
V-RAS	Forward primer (5′- 3′): GGTGGTGTTGGGAAAAGTGC	
	Reverse primer (5′- 3′): CCTTCGCCTGTCCTCATGTA	

C-RAS	Forward primer (5′- 3′): CCCATGGTGTTGGTTGGGAA<br>Reverse primer (5′- 3′): AAAGCATCCTCCACGCCCT
BRCA1	Forward primer (5′- 3′): ATTCACCCCAGACCAGGGTA<br>Reverse primer (5′- 3′): GGGGACCAGATAGGTGTCCA
p16	Forward primer (5′- 3′): GAGCTGCCTACCTCTGAAGTC<br>Reverse primer (5′- 3′): CTCACTAGCCATCAGCACGA
p21	Forward primer (5′- 3′): CTGTGCGCAGATTCACGGAG<br>Reverse primer (5′- 3′): GGCGTCTCGGTGACAAAGTC
p53	Forward primer (5′- 3′): TCTGGGACAGCCAAGTCTGTG<br>Reverse primer (5′- 3′): TTTCCTTCCACTCGGATAAGATG
RAD51	Forward primer (5′- 3′): CCCGACTAGAGCAATGTGGT<br>Reverse primer (5′- 3′): TCGCCTTTGGTGGAACTCAG
RAD52	Forward primer (5′- 3′): GTTGAAACCAGAACCACCGC<br>Reverse primer (5′- 3′): TTGCTCCTAGAACACTCGGC
BRCA2	Forward primer (5′- 3′): CGCGTTTTGTCAGACTCCTC<br>Reverse primer (5′- 3′): GTTGGCCTCTCTTTGCATCC
RAD50	Forward primer (5′- 3′): AGCCTCACTCATCATTCGCC<br>Reverse primer (5′- 3′): GGAAGTTCCGCTGTTGTGAG
MRE11	Forward primer (5′- 3′): AGCTGTTACTCTCCCGCCTA<br>Reverse primer (5′- 3′): TCCTCCGAGACCAGTTCCTT
PARP1	Forward primer (5′- 3′): TTCGATGGGAAAATCCCGCA<br>Reverse primer (5′- 3′): TGCCTGAAACATCTGTCCGT
XRCC1	Forward primer (5′- 3′): CATCTCTTGGGTCAGGCGAA<br>Reverse primer (5′- 3′): TGCCTGAAACATCTGTCCGT
RIF1	Forward primer (5′- 3′): CCCGATCTGGGAAAGTGGAG<br>Reverse primer (5′- 3′): CTGGTCAGGGTCAGGTAAGC
CDK1	Forward primer (5′- 3′): AGCTGGCGCTTGGAAGTTAG<br>Reverse primer (5′- 3′): CTTCATGGCTACCACTTGGC

TERC	Forward primer (5′- 3′): CGCTGTGCTTTTGGTTACCG
	Reverse primer (5′- 3′): GCTGACAGAGCCCAACTCTT
POT1	Forward primer (5′- 3′): ATTTAAAACCCCAGTCTTTCACTTG
	Reverse primer (5′- 3′): CGCTAAACTAACAATGCCTCTAAA
TPP1	Forward primer (5′- 3′): TTCCACGATTCAGACCTGGC
	Reverse primer (5′- 3′): AAGGCTCCTGTGACTCATGC
TRF1	Forward primer (5′- 3′): CAGTCTGCGGTAACTGAATCCT
	Reverse primer (5′- 3′): TCAGTGGCTCGTCTACTGTTC
TRF2	Forward primer (5′- 3′): CGCTGGGTGCTCAAGTTCTA
	Reverse primer (5′- 3′): CCTTCTTCAATGCGCGACAG
TIN2	Forward primer (5′- 3′): GCCATTTCGGAACTCATGCC
	Reverse primer (5′- 3′): TAAGGAGGATCCCTCTCGCT
RAP1	Forward primer (5′- 3′): GATTCTGTGCACCTGCCCC
	Reverse primer (5′- 3′): AAGAGACAGGTAGGTCCCCC
CTC1	Forward primer (5′- 3′): CCGCCTGCTCAGGTACAGA
	Reverse primer (5′- 3′): TCGCAGTTCCATCAGCACATA
STN1	Forward primer (5′- 3′): TCCTCTCTGGAACCCACGTA
	Reverse primer (5′- 3′): TACCTGGCCAGACTCCTTCA
TEN1	Forward primer (5′- 3′): CCGGAAGAAGGAGCAGTAAT
	Reverse primer (5′- 3′): GGATTGGGTGCTGGTCAGAT
PINX1	Forward primer (5′- 3′): TGCGAGTCCGGTAGTTTCAG
	Reverse primer (5′- 3′): TCCTGAGCCCCTAAACCCTT
RIF1	Forward primer (5′- 3′): AGTAAACAGCCCGAACTGGG
	Reverse primer (5′- 3′): TCCAAAGTCTCCAACAGCGG
HoxC5	Forward primer (5′- 3′): ATGAAGCGGCTCCTCTGAAC
	Reverse primer (5′- 3′): AGTCTGGTAGCGCGTGTAAC
C-ABL1	Forward primer (5′- 3′): GGGGAATGTGAAATCCCACG
	Reverse primer (5′- 3′): AAGGGCTTCGTGTTCCACAA

AKT2	Forward primer (5′- 3′): GACGACCCCATGGACTACAAGT<br>Reverse primer (5′- 3′): CGATGATCACCTCCTTCCGCA	
AKT3	Forward primer (5′- 3′): ACCCCAAGATGTGGATTTACCT<br>Reverse primer (5′- 3′): TGTGTTTGGCTTTGGTCGTTC	
PKCα	Forward primer (5′- 3′): GACGAGCTGTTCCAGTCCAT<br>Reverse primer (5′- 3′): TCCTTTGCCGCACACTTTGG	
SRC	Forward primer (5′- 3′): GCTTCAACTCCTCGGACACA<br>Reverse primer (5′- 3′): CCAGTCTCCCTCTGTGTTGT	
SHP-2	Forward primer (5′- 3′): TAATGACCGCTACACCGTCG<br>Reverse primer (5′- 3′): TCTTTTTATCGGCCCCGTCC	
VIMENTIN	Forward primer (5′- 3′): CTCTGAAGCTGCTAACCGCA<br>Reverse primer (5′- 3′): AGGCGGCCAATAGTGTCTTG	
h*TERC*	Forward primer (5′- 3′): TCCACCGTTCATTCTAGAGCA<br>Reverse primer (5′- 3′): ACTCGCTCCGTTCCTCTTC	Primers for RIP assay
h*GAPDH*	Forward primer (5′- 3′): AGCCACATCGCTCAGACACC<br>Reverse primer (5′- 3′): GTACTCAGCGGCCAGCATCG	
g*TERC*	Forward primer (5′- 3′): GGCAGCCATTTCTCATCTAACCCTAA<br>Reverse primer (5′- 3′): CTGACAGAGCCCAACTCTTCACGG	
gβ-actin	Forward primer (5′- 3′): ATCCTGCGGCATTCACGAAA<br>Reverse primer (5′- 3′): GCCAGGGCAGTGATCTCTTT	
mTerc	Forward primer (5′- 3′): TGGTCTTTTGTTCTCCGCCC<br>Reverse primer (5′- 3′): GTTTTTGAGGCTCGGGAACG	
mGapdh	Forward primer (5′- 3′): GTGTTCCTACCCCCAATGTGT<br>Reverse primer (5′- 3′): ATTGTCATACCAGGAAATGAGCTT	
b*TERT*	Forward primer (5′- 3′): ATGCCGCGCGCGCCCAGGTGC<br>Reverse primer (5′- 3′): TCAGTCCAAGATGGTCTTGA	Primers for amplification of TERTs fragment
h*TERT*	Forward primer (5′- 3′): ATGCCGCGCGCTCCCCGCTG<br>Reverse primer (5′- 3′): TCAGTCCAGGATGGTCTTGAAGTC	

mTert	Forward primer (5′- 3′): ATGACCCGCGCTCCTCGTTGCCCCG<br>Reverse primer (5′- 3′): TTAGTCCAAAATGGTCTGAAAGTCT	
b*TERT*-△C	Forward primer (5′- 3′): ATGCCGCGCGCGCCCAGGTGCCGGG<br>Reverse primer (5′- 3′): TCACAGCAGCAGGCCGCACCAGGGG	
h*TERT*-△C	Forward primer (5′- 3′): ATGCCGCGCGCTCCCCGCTGCCGAG<br>Reverse primer (5′- 3′): TCACAGCAGCAGGCCGCACCAGGGG	
m*TERT*-△C	Forward primer (5′- 3′): ATGACCCGCGCTCCTCGTTGCCCCG<br>Reverse primer (5′- 3′): TTACAGCAGCAAGCCACACCAGGGA	
b*TERT*-CTD	Forward primer (5′- 3′): GATACCCGCACCCTGGAGGTGCATG<br>Reverse primer (5′- 3′): TCAGTCCAAGATGGTCTTGAAGTCT	
h*TERT*-CTD	Forward primer (5′- 3′): GATACCCGGACCCTGGAGGTGCAGA<br>Reverse primer (5′- 3′): TCAGTCCAGGATGGTCTTGAAGTCT	
m*TERT*-CTD	Forward primer (5′- 3′): GACACTCAGACTTTGGAGGTGTTCT<br>Reverse primer (5′- 3′): TTAGTCCAAAATGGTCTGAAAGTCT	
SV40LT	Forward primer (5′- 3′): ATGGATAAAGTTTTAAACAGAGA<br>Reverse primer (5′- 3′): TTATGTTTCAGGTTCAGGGGG	Primers for amplification of *SV40LT* and *PINX1* fragment
b*PINX1*	Forward primer (5′- 3′): GCTGCCGTTTCCCCGGCGTGCGAGT<br>Reverse primer (5′- 3′): TTTTTTTTTTTTTTTTCTAGTAAGG	
TS primer	5′- 3′: AATCCGTCGAGCAGAGTTAGGGTTAG	Primers for telomeres activity assay
CX primer	5′- 3′: CCCTTACCCTTACCCTTACCCTA	
Telo	Forward primer (5′- 3′): CGGTTTGTTTGGGTTTGGGTTTGGGTTTGGGTTT<br>Reverse primer (5′- 3′): GGCTTGCCTTACCCTTACCCTTACCC	Primers for relative telomeres length
gβ-globin	Forward primer (5′- 3′): CCCTGCCCTTGCTTAATGTC<br>Reverse primer (5′- 3′): CAGGAAGGGGAGCTTAGTGA	
bβ-globin	Forward primer (5′- 3′): GTGTTTTGAGCATCTGGCCTC<br>Reverse primer (5′- 3′): AGGTCTCGACTAGCCCTTCAT	
mβ-globin	Forward primer (5′- 3′): GTGCTGGTGATTGTTTTGGCT<br>Reverse primer (5′- 3′): CGATTTTGATTCGGAGGTGCT	

sgRNA1	Top Guide oligo (5′- 3′): CACCGTGCCTGAAACTCGCGCCGCG<br>Bottom Guide oligo (5′- 3′): AAACCGCGGCGCGAGTTTCAGGCAC	Primers for sgRNA cloning
sgRNA2	Top Guide oligo (5′- 3′): CACCGACGTAGAGCCCGGCGTGACA<br>Bottom Guide oligo (5′- 3′): AAACTGTCACGCCGGGCTCTACGTC	
sgRNA3	Top Guide oligo (5′- 3′): CACCGGTCGAGTCTCGATTATGGGC<br>Bottom Guide oligo (5′- 3′): AAACGCCCATAATCGAGACTCGACC	
sgRNA4	Top Guide oligo (5′- 3′): CACCGTCGTACCTGGGCGCAAACAC<br>Bottom Guide oligo (5′- 3′): AAACGTGTTTGCGCCCAGGTACGAC	
sgRNA8	Top Guide oligo (5′- 3′): CACCGCATCCTCGACCATTCCGCAG<br>Bottom Guide oligo (5′- 3′): AAACCTGCGGAATGGTCGAGGATGC	
h*TERT*-KO	Forward primer (5′- 3′): ATTCGCGGGCACAGACGCCCAGGAC<br>Reverse primer (5′- 3′): GAGTGGAAGCCGGGCTCCTGGTGAGGA	Primers for amplification of knock-out target sites
b*TERT* pro-KO	Forward primer (5′- 3′): ACGGAAGATCCCGCAAAGAGAAAG<br>Reverse primer (5′- 3′): GCGCGGAGGAGCAGGGGTCCGGGC	

Table S2. Sequences of primers for Junction PCR

Primer	Sequence (5′-3′)	Product length
Lj-F	CGGCGCGAGCTGCAATCCTGAGG	1030 bp
Lj-R	TCCCACCGTACACGCCTACC	
Rj-F	ATCCACCGGATCTAGATAACTGATC	1316 bp
Rj-R	GGAGTTGGATCGGTTACTTCCAT	

Table S3. Sequences of sgRNA3 potential on/off target sites

Name	On-target site	potential off-target site
sgRNA3-OT1	GTCGAGTCTCGATTATGGGCGGG	ACTTTCTGAGTCTCGATTATGGGC
sgRNA3-OT2		CCAGCCCACAATAGAGACTCAAC
sgRNA3-OT3		CTGGTCCAGCCCACAATAGAGAC
sgRNA3-OT4		AAAAAGCACAATAGAGACTCAAC
sgRNA3-OT5		TGTTTATTCAGCCCACAATAGAGA
sgRNA3-OT6		CATAGAACACAATAGAGACTCAA
sgRNA3-OT7		GTTATAAACAATAGAGACTCAAC
sgRNA3-OT8		ATTGCATACAATAGAGACTCAAC

Table S4. Primer sequences for amplification of potential off target region.

Name	Primer	Sequence (5′-3′)	Product length
sgRNA3-OT1	Forward	GGGTACTGTTAAAAA-	617 bp
	Reverse	CCTGTATCCTTTTTCTCTGCTTATTCAAAC	
sgRNA3-OT2	Forward	CTGTGTCAGAGATAAAGAGGG-	623 bp
	Reverse	TACACTCTCCCACGCATGTGGCAGTATATG	
sgRNA3-OT3	Forward	CACCGGGCTACCCCCCCCACTA-	618 bp
	Reverse	ACAGGGGGCTCCCCGGCTCTCACCGTCCC	
sgRNA3-OT4	Forward	GAAATGTTCTATATTTTGCTTGGGCTG	617 bp
	Reverse	AGTTAACCAGGAATGCATTTAATGATGGC	
sgRNA3-OT5	Forward	GTAGATGCTGTGGCCTGCAGTTATCAACC	616 bp
	Reverse	GCCTTCATTCTCTGGGACAGTCCTCTC	
sgRNA3-OT6	Forward	AAAACAGGTAATGAATTAGAGGAGAG	616 bp
	Reverse	CTTTAATCCAATTTTCTACTGATGGATG	
sgRNA3-OT7	Forward	ACTCCAGAGTCTTAGTGATTCTCAGG	616 bp
	Reverse	ACATCAGGTAACCAAAATATTGGAGCTTC	
sgRNA3-OT8	Forward	ACATAGACGTGTCATTTGGAGCGGCAATG	579bp
	Reverse	ATGAGATGGTTGGATGGCATCAC-	

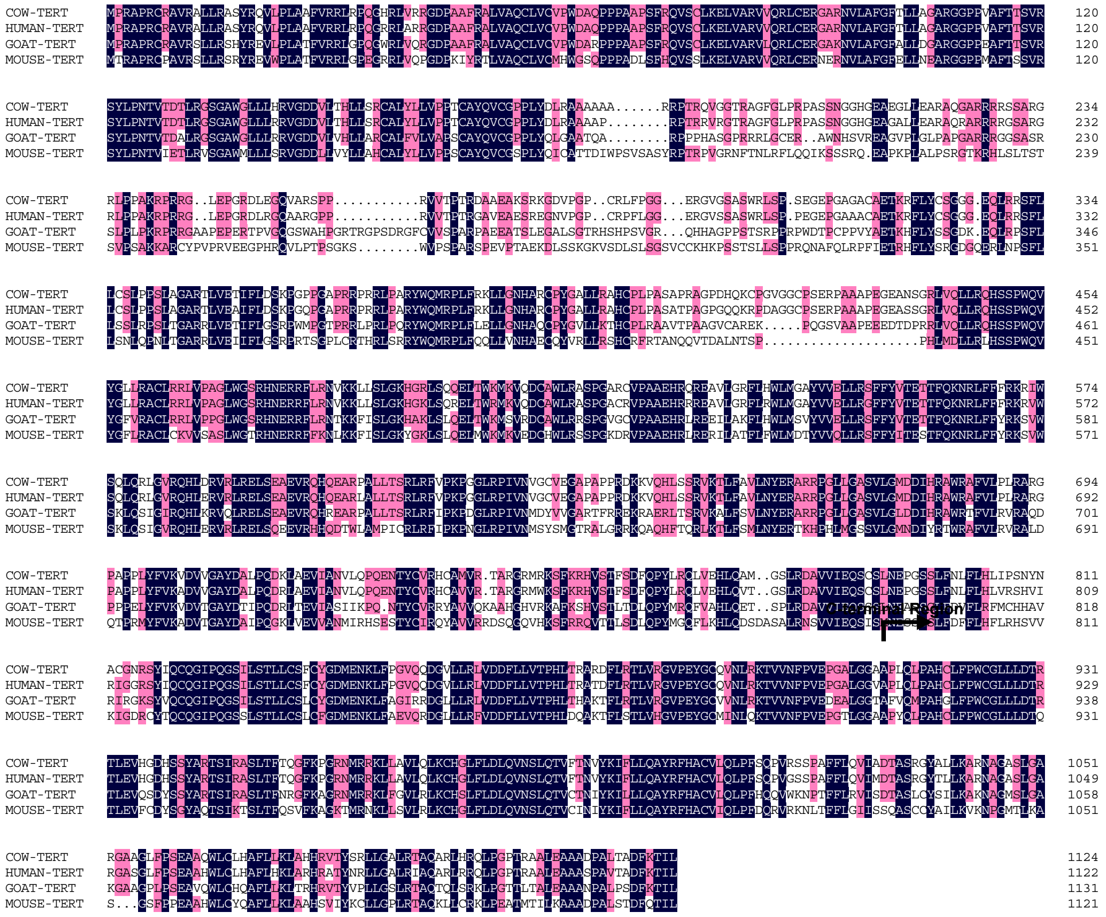

Figure S1. Schematic representation of alignment of bovine, human, goat and mouse TERT protein sequences. Sequence alignment of the C-terminal amino acid sequences of four TERTs. Mammalian TERTs protein sequences are aligned to show 100% (dark blue) and ≥75% (magenta) conserved amino acids through DNAMAN.

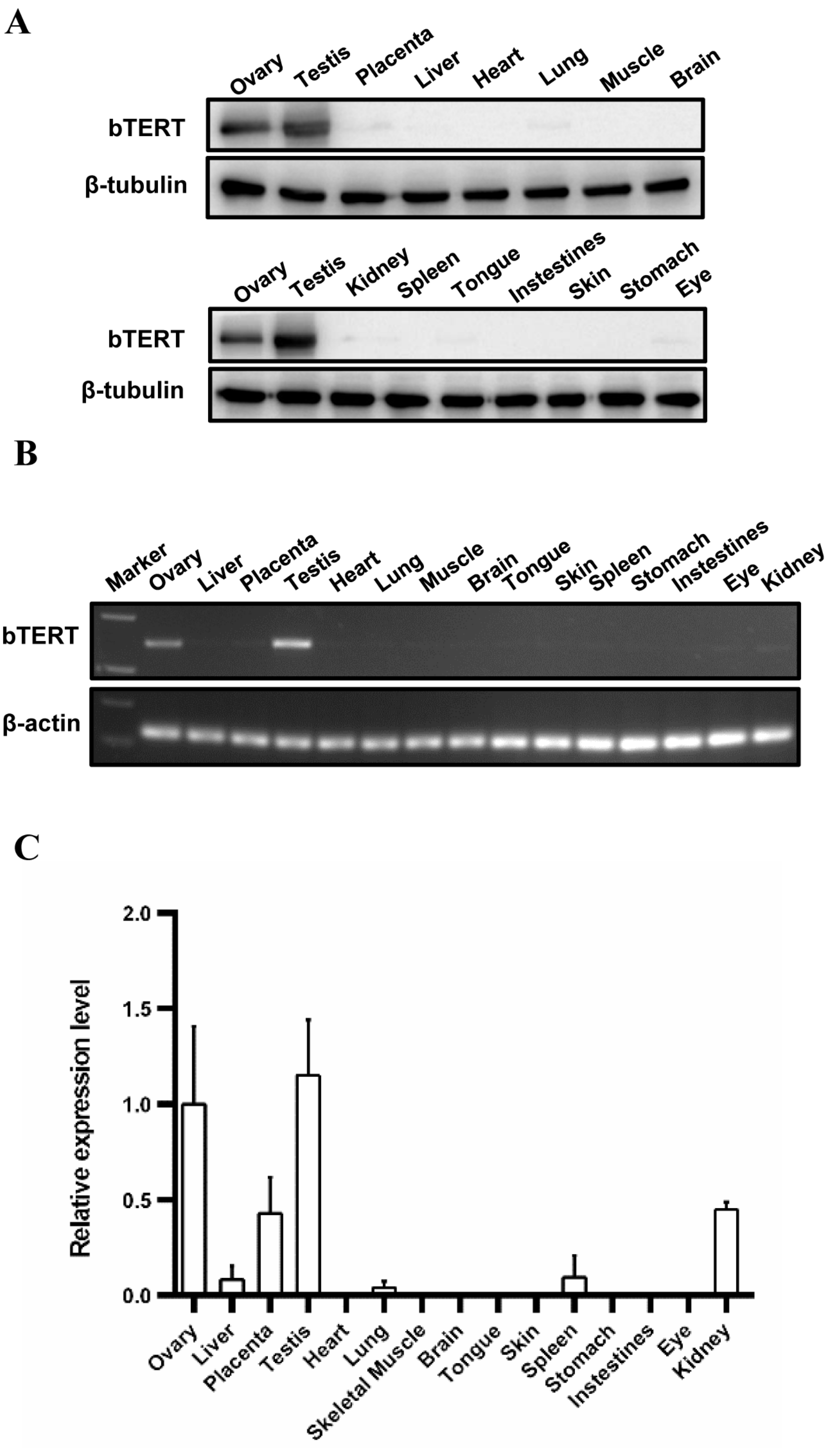

Figure S2. Expression of bTERT in different tissues. Expression level of bTERT was examined by Western blot **(A)**, semi-quantitative PCR **(B)** and qPCR **(C).** Bovine β-actin served as internal reference, n = 3 independent experiments.

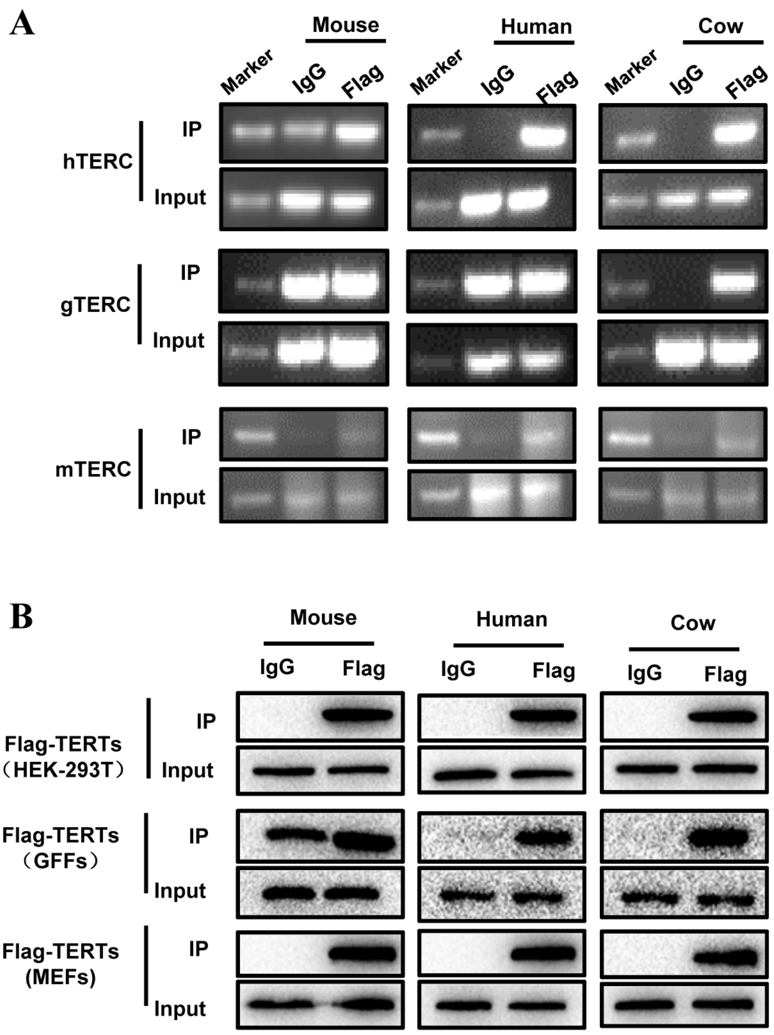

Figure S3. The ability of TERTs interacting with TERCs in cells of different species. HEK-293T, GFFs or MEFs were transfected with Flag-tagged mTERT, hTERT or bTERT respectively. Flag-tagged TERTs were immunoprecipitated respectively and precipitates were detected by semiquantitative PCR for TERCs **(A).** Flag-tagged TERT proteins were immunoprecipitated respectively and precipitates were blotted for three TERT proteins from different species. Total cell lysate (input) was set as internal reference for RNA binding protein immunoprecipitation assays and normal IgG served as a negative control. The immunoprecipitation efficiency of the bait protein was shown in **(B)**.

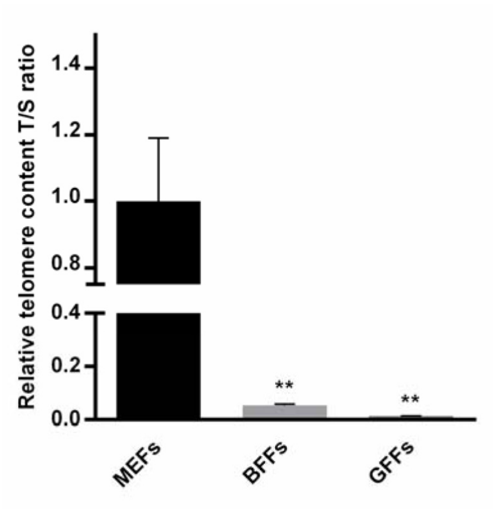

Figure S4. Relative telomere length of primary MEFs, BFFs and GFFs. Bovine β-globin served as internal reference. Data were mean ± s.d., $n = 3$ independent experiments, one-tailed Student's t-test. ** $p < 0.01$ vs MEFs.

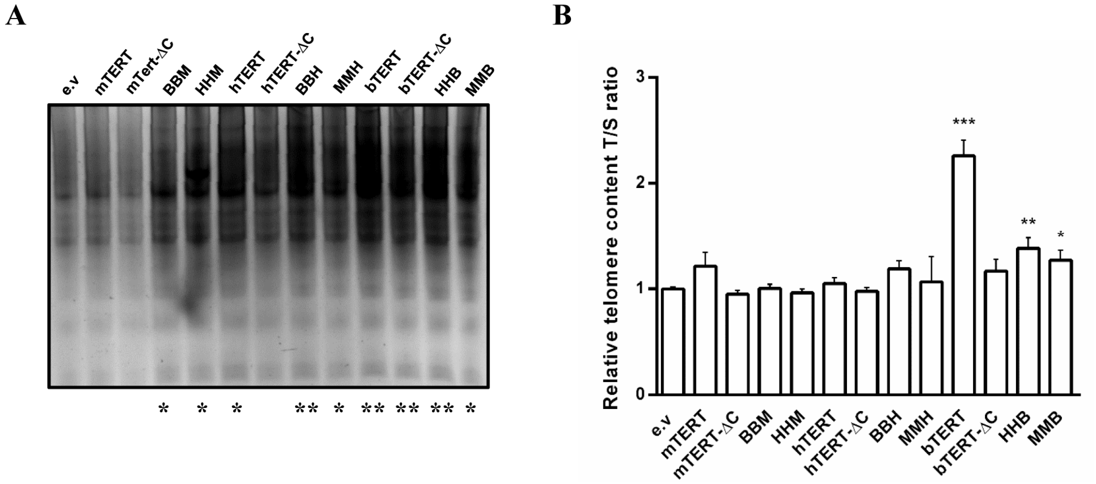

Figure S5. The telomerase activity and relative telomere length of GFFs transfected with *TERT* mutants. The telomerase activity **(A)** and relative telomere length **(B)** of *TERT*s, truncated expression vectors of TERTs without CTD domain, the substitute recombinant expression vectors of TERTs with CTD replacement of TERTs of different species or empty vector transfected in GFFs respectively. e.v, empty vector. Data were mean ± s.d., $n = 3$ independent experiments, one-tailed Student's t-test, * $p < 0.05$, ** $p < 0.01$, ***$p < 0.001$ vs. e.v.

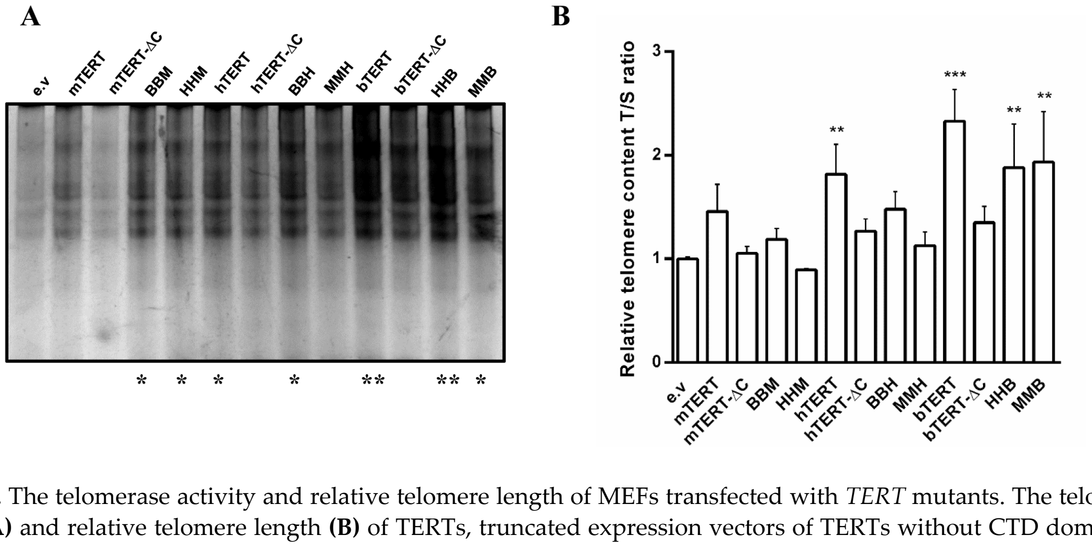

Figure S6. The telomerase activity and relative telomere length of MEFs transfected with *TERT* mutants. The telomerase activity **(A)** and relative telomere length **(B)** of TERTs, truncated expression vectors of TERTs without CTD domain, the

substitute recombinant expression vectors of TERTs with CTD replacement of *TERT* of different species or empty vector transfected in MEFs respectively. e.v, empty vector. Data were mean ± s.d., n = 3 independent experiments, one-tailed Student's t-test, * $p < 0.05$, ** $p < 0.01$, *** $p < 0.001$ vs. e.v.

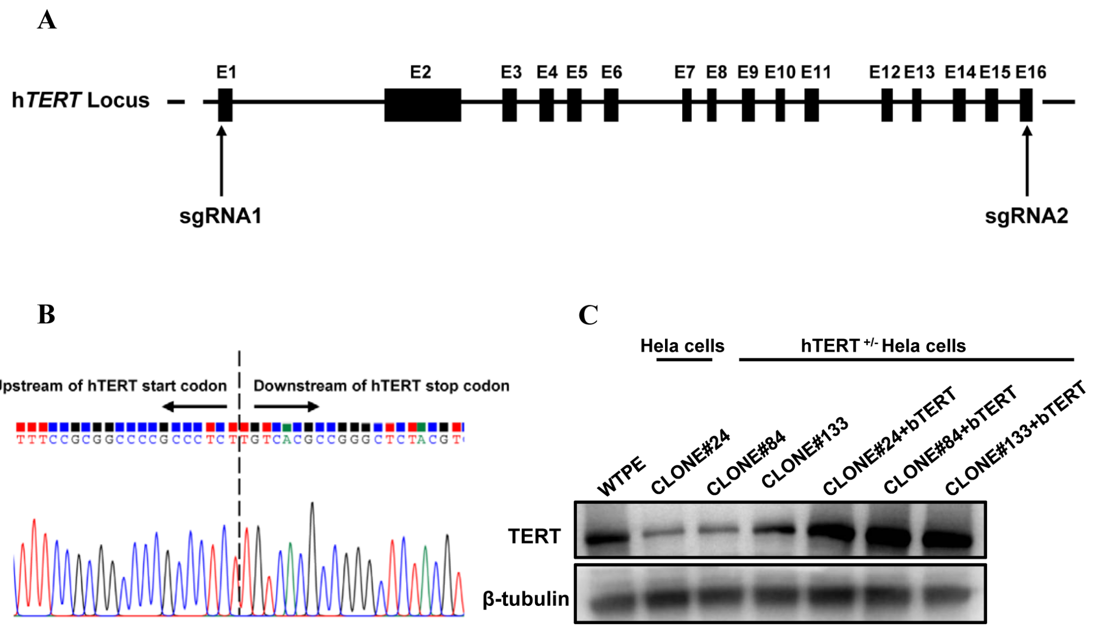

Figure S7. hTERT deletion via CRISPR/Cas9 system in Hela cells. **(A)** Design illustration of Cas9 gRNAs (sgRNA1 and sgRNA2) targeting h*TERT* in Hela cells. E, Exon. **(B)** Sanger sequencing results of target site of h*TERT* $^{+/-}$ Hela colonies. **(C)** Expression of TERT in WTPE, three h*TERT* $^{+/-}$ colonies of Hela cells (CLONE#24, CLONE#84 and CLONE#133), as well as these three h*TERT* $^{+/-}$ colonies resupplied with bTERT by Western blot. "WTPE" represents wild-type Hela cells. Human β-tubulin served as internal reference.

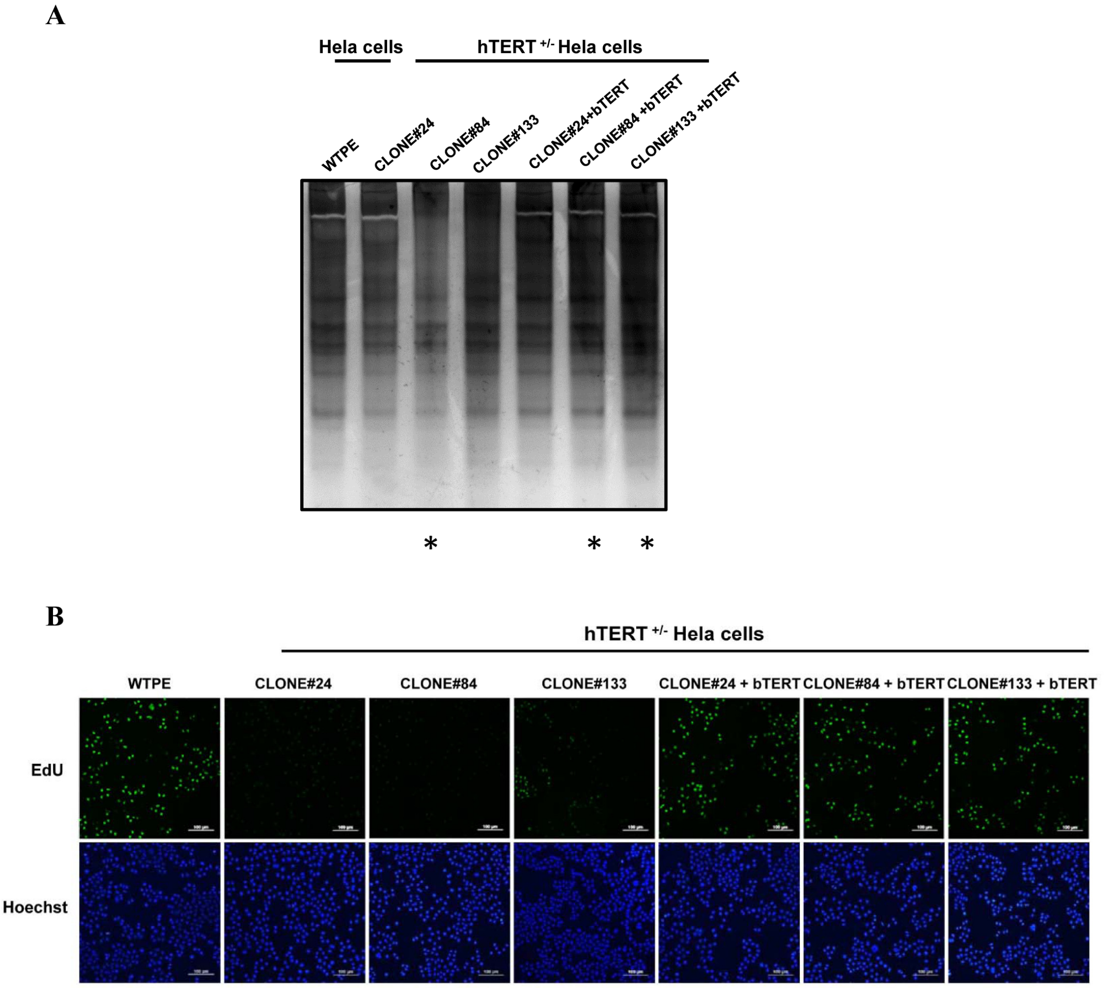

Figure S8. The cell viability and telomerase activity of h*TERT* $^{+/-}$ Hela colonies after resupplying with bTERT. **(A)** Trap analysis and relative intensity analysis of WTPE, three h*TERT* $^{+/-}$ colonies of Hela cells (CLONE#24, CLONE#84 and CLONE#133), as well as these three h*TERT* $^{+/-}$ colonies resupplied with bTERT. "WTPE" represents wild-type Hela cells. Data were mean ± s.d., n = 3 independent experiments, one-tailed Student's t-test, *p≤0.05. vs. WTPE. **(B)** EdU incorporation assay of WTPE, three h*TERT* $^{+/-}$ colonies of Hela cells (CLONE#24, CLONE#84 and CLONE#133), as well as these three h*TERT* $^{+/-}$ clones resupplied with bTERT, and stained with corresponding secondary antibody by Alexa Fluor Plus 488. Each sample was counterstained with Hoechst to visualize DNA (blue) (Scale bars = 100 μm). "WTPE" represents wild-type Hela cells.

A

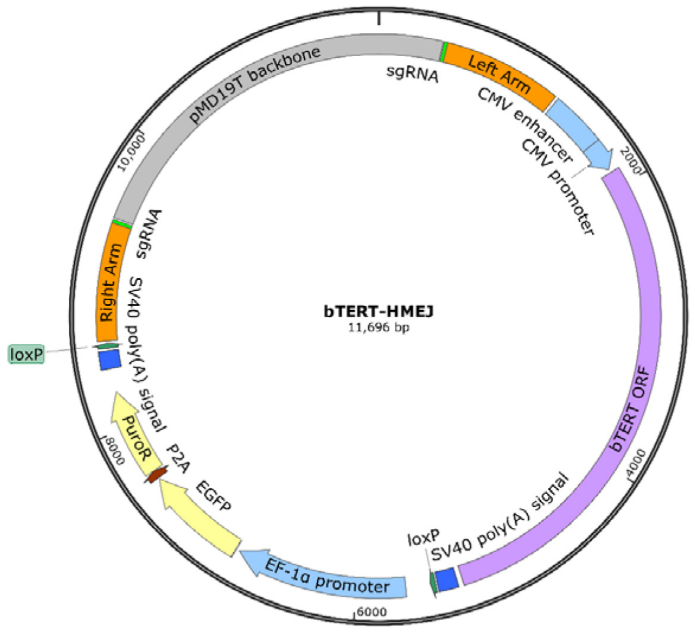

B

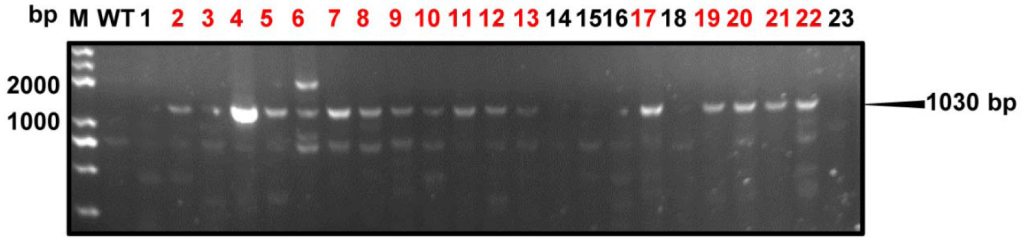

C

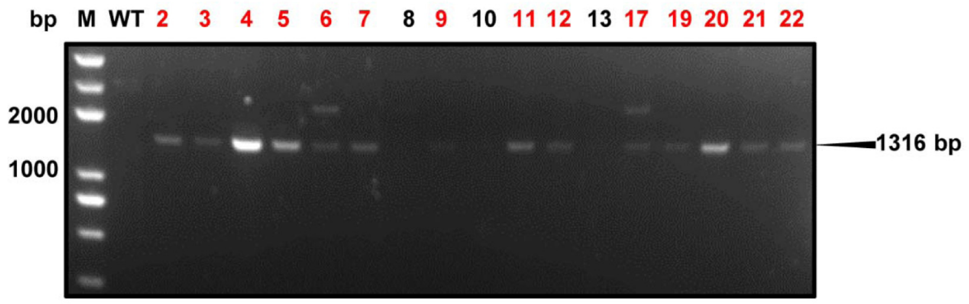

Figure S9. Schematic representation of HMEJ-mediated b*TERT* gene targeting vector and representative junction PCR results of puromycin-resistant colonies. **(A)** Schematic representation of the HMEJ-mediated gene targeting vector. **(B, C)** Representative 3′ junction (B, 1030-bp), 5′ junction (A, 1316-bp) PCR results of puromycin-resistant colonies. Red fonts represent positive results. "WT" represents wild-type cells (non-transfected BFFs). M, marker.

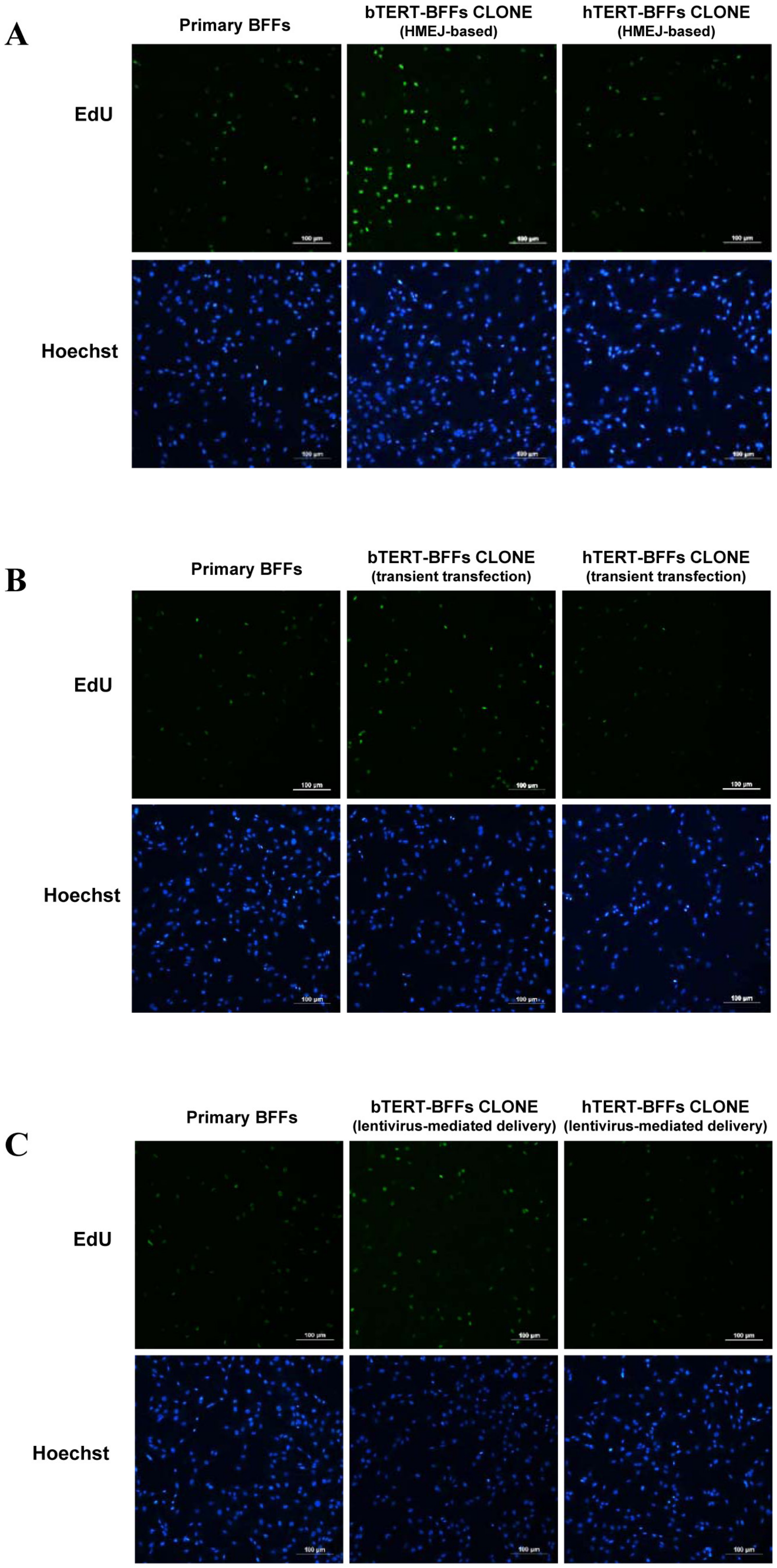

Figure S10. Cell proliferation activity of BFFs colonies generated by different immortalization strategies. EdU incorporation assay of wild-type BFFs, 80th- or 50th-passage of BFFs colonies integrated with b*TERT* or h*TERT* by CRISPR/Cas9 system respectively **(A)**, 15th- or 5th-passage of BFFs colonies via transient transfection with b*TERT* or h*TERT* respectively

(B) and 50th- or 35th-passage of BFFs colonies via lentivirus-mediated delivery with b*TERT* or h*TERT* respectively **(C)**, and stained with corresponding secondary antibody by Alexa Fluor Plus 488. Each sample was counterstained with Hoechst to visualize DNA (blue) (Scale bars = 100 μm).

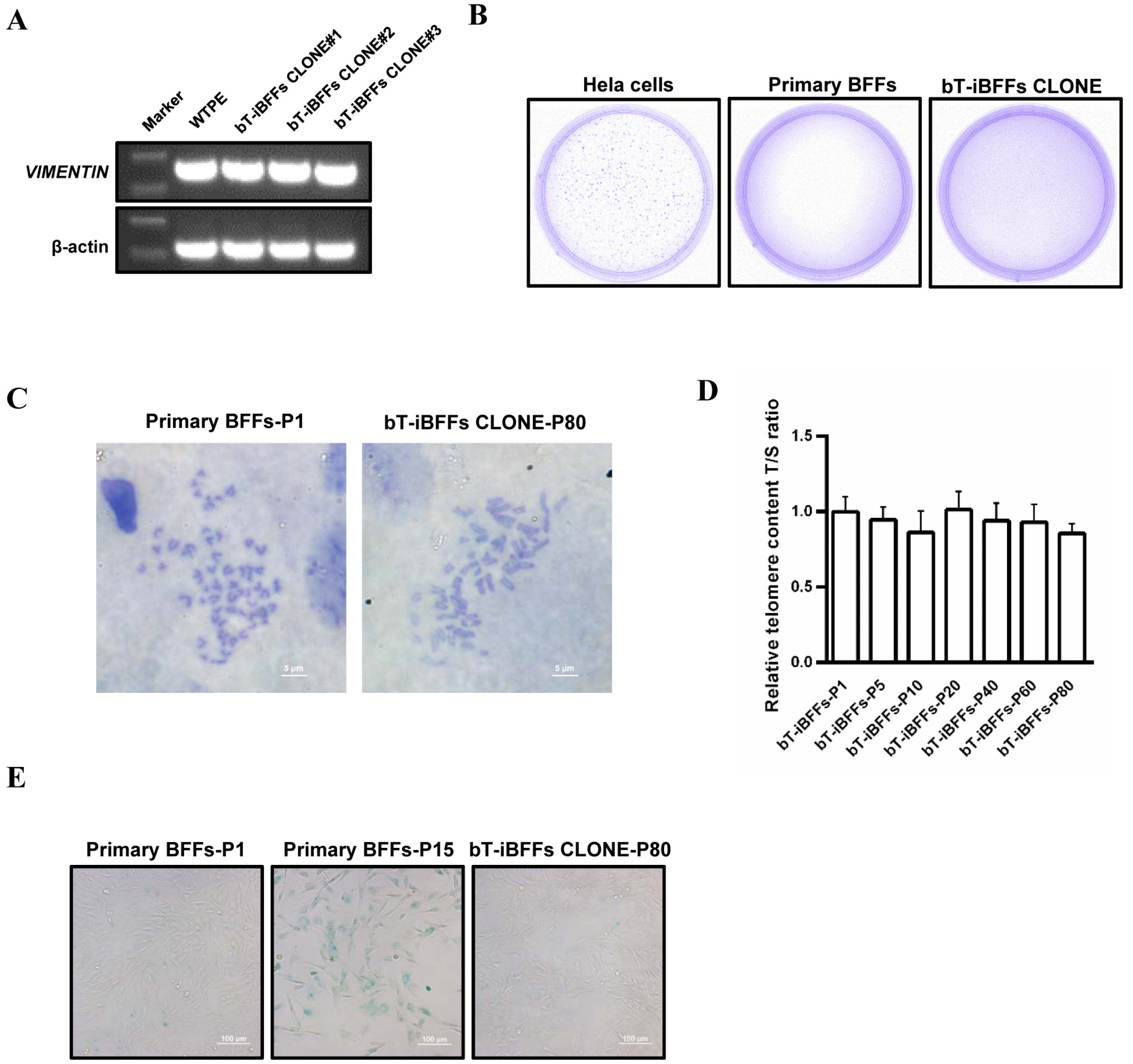

Figure S11. Characteristics of bT-iBFFs by HMEJ-based CRISPR/Cas9-mediated precise integration of b*TERT* into b*ROSA26* locus. **(A)** The expression of *VIMENTIN* of bT-iBFFs colonies and wild-type BBFs by semiquantitative PCR. "WTPE" represents wild-type BFFs. Bovine β-actin served as internal reference. **(B)** Relative telomere length of 1st-, 5th-, 10th-, 20th-, 40th-, 60th-, 80th-passage of bT-iBFFs colony. P1, P5, P10, P20, P40, P60, P80 represents the passage of 1, 5, 10, 20, 40, 60, 80 of the colony respectively. Bovine β-globin served as internal reference. Data were mean ± s.d., n = 3 independent experiments, one-tailed Student's t-test, vs. 1st-passage of bT-iBFFs colony. **(C)** Karyotype analysis of primary BFFs and bT-iBFFs (Scale bars = 5 μm). **(D)** The risk of malignant transformation of bT-iBFFs colony by soft agar assay. Hela cells served as positive control, primary BFFs as negative control. **(E)** The senescence of 80th-passage of bT-iBFFs colony by β-galactosidase staining. The 1st-passage of primary BFFs served as positive control, the 15th-passage of primary BFFs served as negative control. (Scale bars = 100 μm).

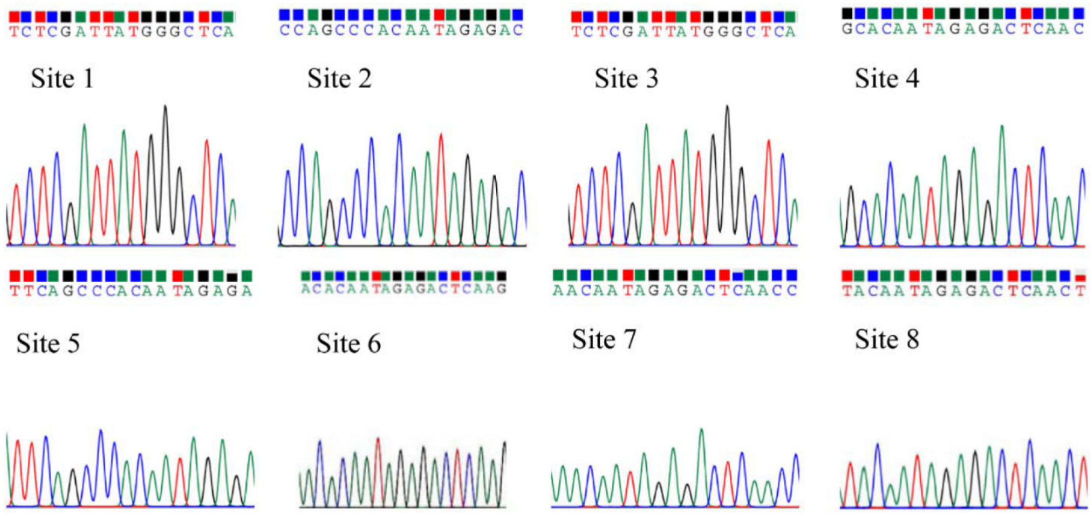

Figure S12. Sanger sequencing results of eight potential off-target sites in bT-iBFFs.

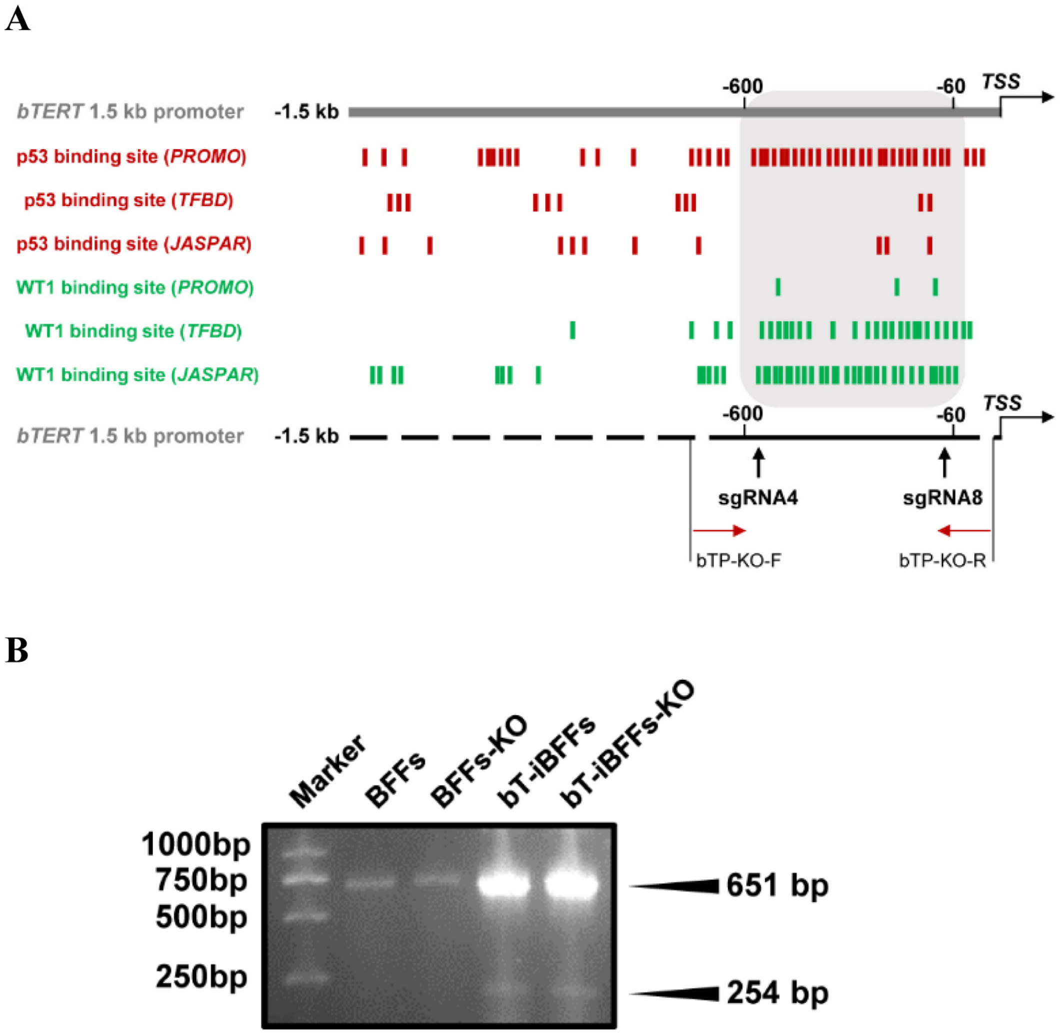

Figure S13. The efficiency of bT-iBFFs for the second genome editing. **(A)** Schematic layout of p53 and WT1 binding sites of the bTERT 1.5 kb promoter predicted by PROMO, TFBD and JASPAR prediction tools. Red for p53 predicted binding site, green for WT1 predicted binding site. Bottom: illustration of gRNAs (sg4 and sg8) targeting bTERT promoter. TSS, transcription start site. BTP-KO-F/BTP-KO-R: PCR primer for amplifying the target sequence of precise knock-out. **(B)** The PCR analyses confirming the precise knock-out in wild-type BFFs and bT-iBFFs.

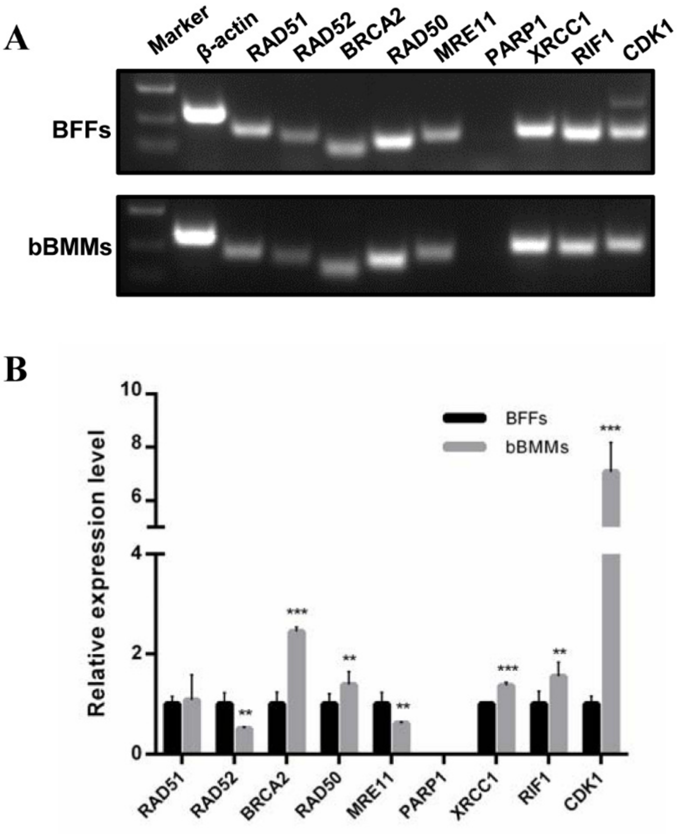

Figure S14. Endogenous expression of crucial factors related to HR pathway and NHEJ pathway in BFFs and bBMMs by semiquantitative PCR (A) and qPCR (B). Bovine β-actin served as internal reference. Data were mean ± s.d., $n = 3$ independent experiments, one-tailed Student's t-test. ** $p < 0.01$, *** $p < 0.001$ vs BFFs.

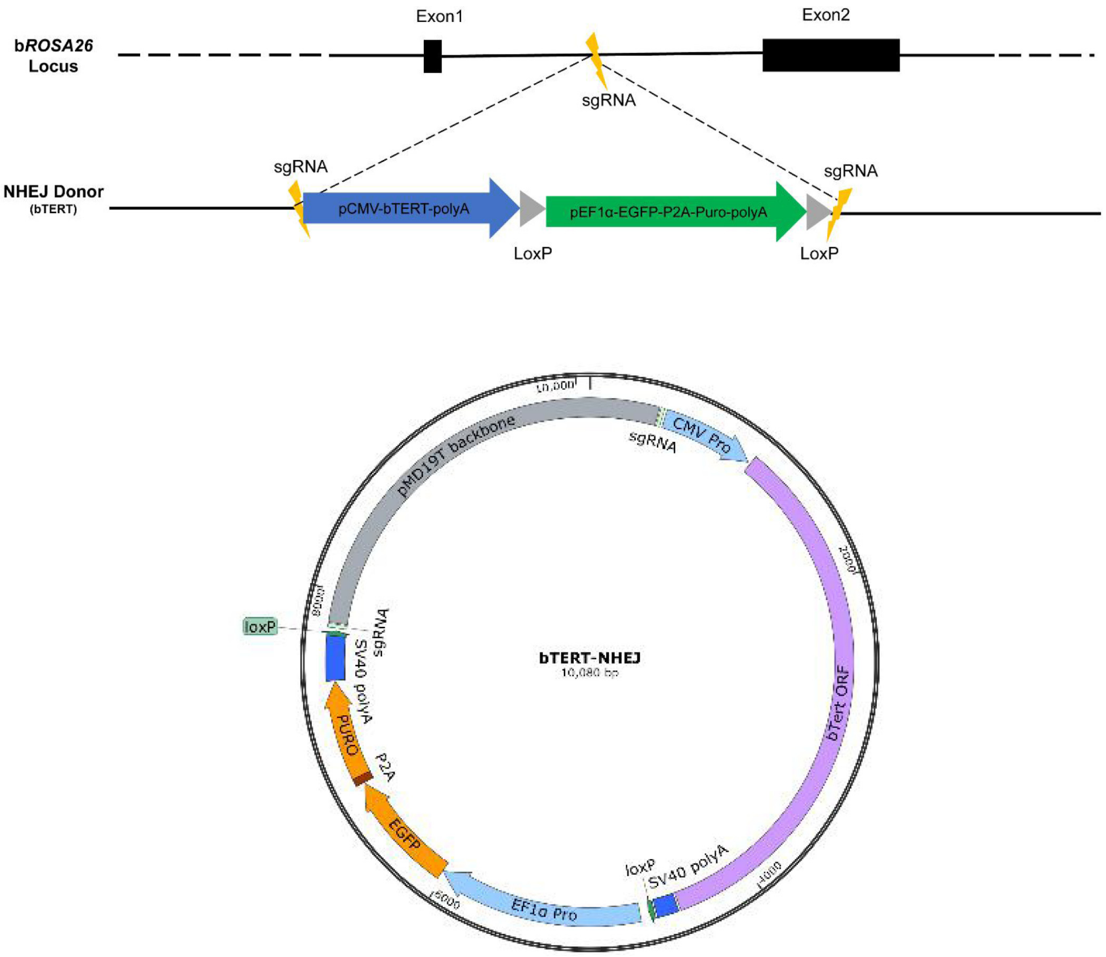

Figure S15. Schematic representation of NHEJ-based precise integration of b*TERT* into b*ROSA26* locus.

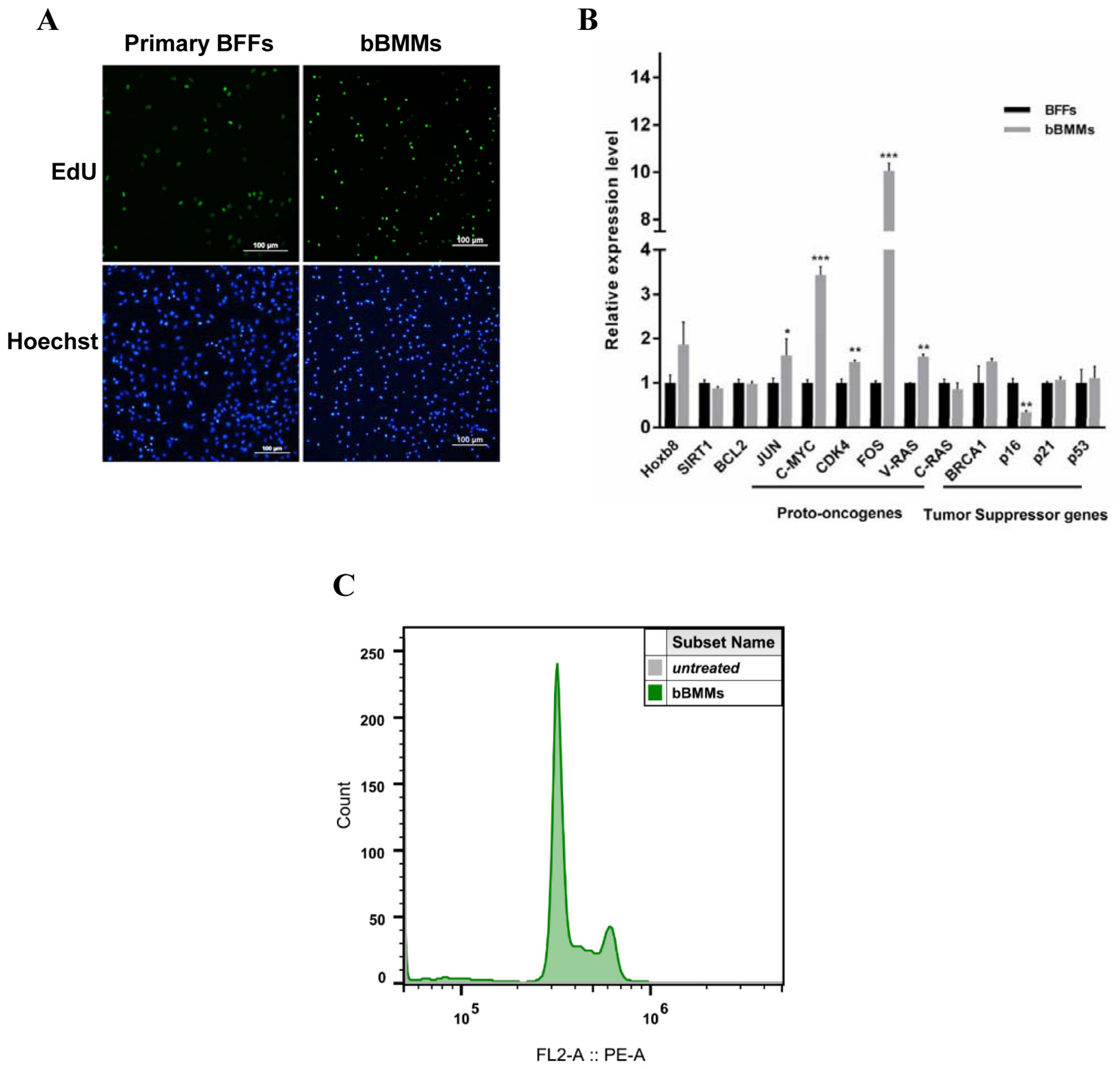

Figure S16. The cell viability and cell cycle of primary BFFs and bBMMs. (A) EdU incorporation assay of primary BFFs and bBMMs, and stained with corresponding secondary antibody by Alexa Fluor Plus 488. Each sample was counter-stained with Hoechst to visualize DNA (blue) (Scale bars=100 μm). (B) Endogenous expression of proliferation-, senescence- and apoptosis-related genes in BFFs and bBMMs. Bovine β-actin served as internal reference. Data were mean ± s.d., $n = 3$ independent experiments, one-tailed Student's t-test. * $p < 0.05$, ** $p < 0.01$, *** $p < 0.001$ vs BFFs. (C) Cell cycle of primary bBMMs by FCAS.

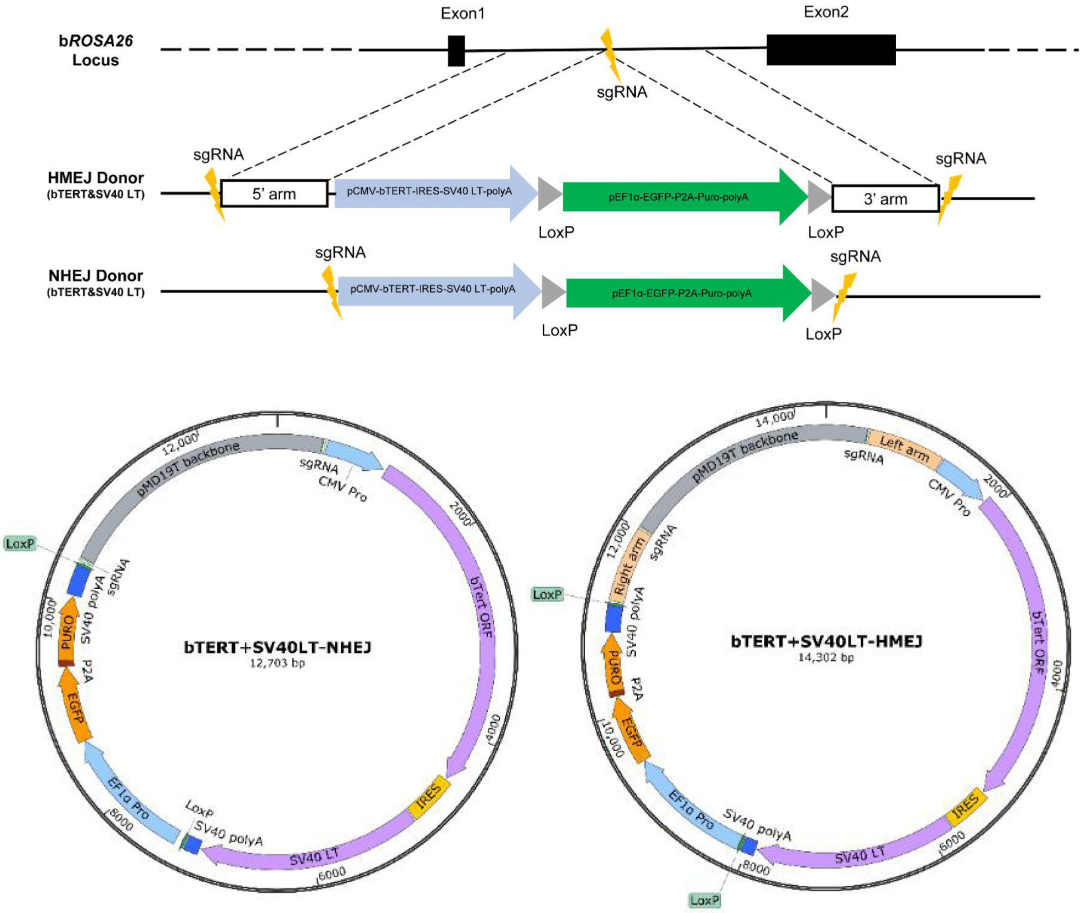

Figure S17. Schematic representation of the NHEJ- and HMEJ-based precise integration of b*TERT* and *SV40LT* into b*ROSA26* locus.

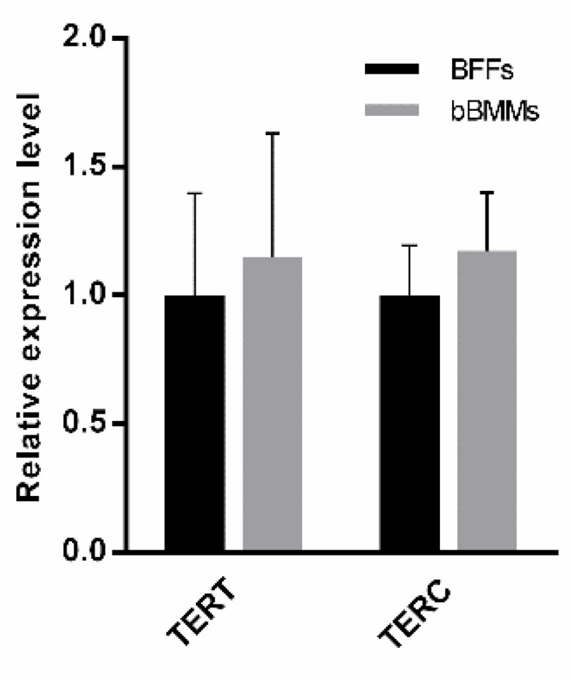

Figure S18. Endogenous expression of b*TERT* and b*TERC* in primary BFFs and bBMMs. Bovine β-actin served as internal reference. Data were mean ± s.d., n = 3 independent experiments, one-tailed Student's t-test, vs BFFs.

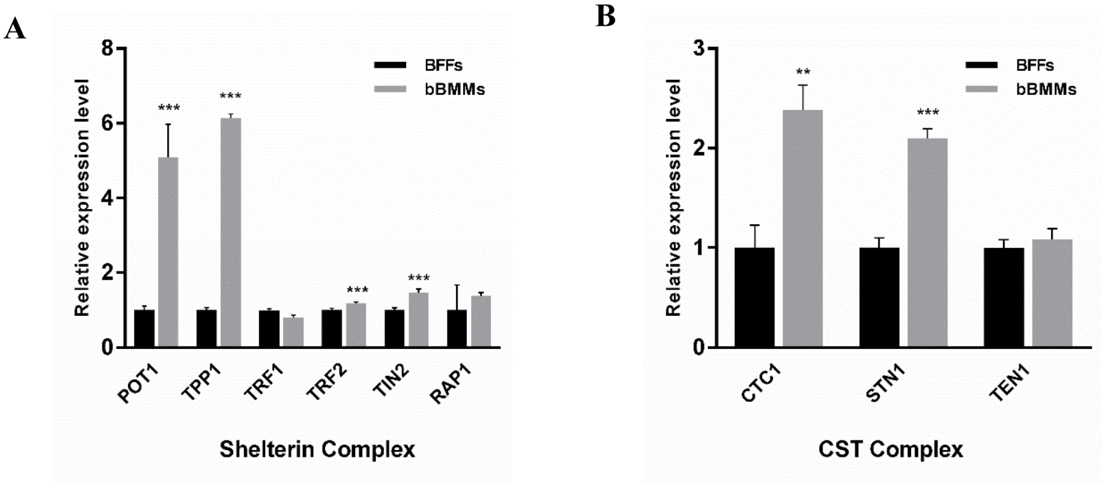

Figure S19. Endogenous expression of components of Shelterin complex (A) and CST complex (B) in primary BFFs and bBMMs. Bovine β-actin served as internal reference. Data were mean ± s.d., $n = 3$ independent experiments, one-tailed Student's t-test. ** $p < 0.01$, *** $p < 0.001$ vs BFFs.
